# Translation of Henrich Klebahn's 'Damaging agents of the klippfish - a contribution to the knowledge of the salt-loving organisms'

**DOI:** 10.1186/1746-1448-6-7

**Published:** 2010-06-22

**Authors:** Priya DasSarma, Günther Klebahn, Helga Klebahn

**Affiliations:** 1 Department of Microbiology and Immunology, University of Maryland School of Medicine, 701 East Pratt Street, Baltimore, Maryland, USA

## Abstract

**Background:**

Henrich Klebahn was a German linguist, mycologist and phytopathologist, who was known as Dr. Dr. h. c. Henrich Klebahn, Hauptcustos a. D., Honorarprofessor an der Hanischen Universität. He was born February 20, 1859 in Bremen, and died October 5, 1942 in Hamburg. He taught linguistics from 1885-1899, studied Natural Science at the Universities of Jena and Berlin (1881) and received his PhD from the University of Jena. In 1899, he was appointed scientific assistant at the Hamburg botanical garden, where he worked until 1905. From 1905 to 1930, he was at the agricultural institute of Bromberg. In 1921, he was named honorary professor and lecturer in cryptogams and soil biology at the Institut für Allgemeine Botanik where he taught until 1934. He is well known for his work on gas vesicles and halophiles, among other topics.

This re-print of 'Die Schädlinge des Klippfisches. Ein Beitrag zur Kenntnis der salzliebenden Organismen. Von H. Klebahn. Mit zwei Tafeln und vier Abbildungen im Text.' was originally published in 1919 in the Jahrbuch der Hamb. Wissensch. Anstaltes. XXXVI. Beiheft pages 11-69, by Latcke & Waltt, E. H. Buchdrucker. The translators have tried to remain faithful to the contents and to the original sense of the article by minimizing modifications.

**Results:**

The original paper reported the conclusions of a 3 year long study of the microbes causing damage to the fish industry as well as a summation of work on the subject up until 1919. The findings were that the causative agents were fungi and other microbes, the chief of which was a red, Gram-negative rod-shaped bacillus, *Bacillus halobius ruber*, that formed pale reddish colonies and was found to oscillate, but after extensive testing, was found not possess flagella. The initial appearance of "a shiny corpuscle" at the ends of cells was determined not to be spores; rather that it was the "result of the coherence of the light beams due to a total reflection of the light in the optically denser little rods". The cells were osmotically sensitive to the addition of water. In addition, a Gram-negative, red *Sarcina morrhuae *that appeared pinker in color, was less salt-sensitive than the red bacillus, in fact surviving the transfer to water. These were "round individual cells or groups of only two or four cells, usually; however, there are eight or more round cells that are arranged like cube corners to great cube-like or irregular packages lying together, just in the same manner as with the familiar *Sarcina ventriculi*." This organism was also identified from the walls of a fish storage room. Finally, a third, red microorganism was isolated: a Gram-negative micrococcus, *Micrococcus (Diplococcus) morrhuae*, which was "spherically rounded" and barely sensitive to water: "If one distributes a sample of a colony in water, the cells partly separate, to a great degree; however, they stay together in groups of two or four cells."

**Conclusions:**

This article provides evidence for identification of halophilic microbes as the major cause of fish spoilage, and is one of the earliest publications in the field of halophile microbiology.

## Introduction

The following description is the temporary conclusion of research and experimentation that took place in response to the encouragement by the fish directorate in Hamburg, which was seeking means to fight the fungi and bacteria that attack the klippfish during its preparation and storage. The organisms have been a great plague to the fish industry and fish stores for many years, which they have not been able to master despite much effort. My work, which began in the summer of 1916, and specifically, the attempts at fighting the evils, suffered from the cessation of the entire fish import in the year 1919, led to an unwelcome interruption, and has since not been able to be resumed. The scientific research has led to a number of results, which though they certainly cannot be considered completed, do still permit a momentary summary and description. These will be given together with a historical overview about the knowledge of the damaging agents.

I extend my warmest thanks to Herr Fishereidirektor Duge (Hamburg), Dr. med. Duge (Cuxhaven), business leader Klockau (klippfish works Cuxhaven), Prof. Dr. B. Walter (Physical State Laboratory), Prof. Dr. W. Göhlich (Chemical State Laboratory), Prof. Dr. H.C. Plaut (Institute for Fungal research at the Eppendorfer Hospital), Dr. C. Schwarze (Institute for General Botany), as well as the administration of the Institute of Hygiene, especially Prof. Dr. W.P. Dunbar, Prof. Dr. J. Kister and Dr. O. Kammann for the valuable encouragement of my work.

A few notes about the production of the klippfish should be provided first. The raw fish that are prepared for the production are basically *Gadus morrhua *(cod, Dorsch), *Gadus virens *(bluefish, Köhler), *Gadus pollachius *(pollack) and *Brosmius brosme *(lub). After removal of the head and the intestines of the fish, and the accomplishment of a skillful lengthwise cut that renders possible a flat spreading of the individual animal, the fish are salted. This usually is done simply by stacking them alternately with thick enough layers of table salt into large heaps. The completely salted fish, which can be stored for lengthy periods of time, or even be sent out (as salt fish) are dried later. According to the original method that is still common in Norway, one dries them on cliffs with the air and sun (klippfish, or cliff fish). In recent times one does the drying in factories. There are several patented methods, all of which have in common that they use artificially dried air. In Germany, there were two factories in operation in 1916, one in Geestemünde that employed over 900 ladies, and a smaller one in Cuxhaven. They basically dried the salt fish from Norway. In Hamburg, there was a large fish warehouse, where over 400 ladies were employed. The newly arrived fish, which often is already attacked by pests, is brushed and washed with water in wooden troughs and then again layered with salt, all before drying. Even though, initially, it would appear as though the pests were removed by this procedure, it stands to reason to any person knowledgeable about fungi, that the pests could have simply been temporarily delayed in their development. Since the water is not continuously renewed, the possibility even exists that the germs are simply more evenly distributed over all the surfaces of the fish. Only if the fish is completely dried, can the danger be considered largely overcome, but only as long as it stays dry. If it takes up damp air or water, and that happens both in layering of the fish again as well as in the commercial shipping, the pest growth can start anew.

## I. Torula epizoa

One of the pests of the klippfish is a fungus from the Dematiacee group, which was first observed by Corda [1] on salted meat and described as *Torula epizoa *(1829). Later, Kickx [2,3] described a living specimen as var. *muriae *(1867) from an anchovy and on the infiltrating brine, in which this fish was preserved. The diversity of the two forms seems doubtful. The color of the main type is given as brown, and the variant as earth-grey; the conoid chains for the former are supposed to have bigger conoids from section to section, those of the variant are supposed to be a little smaller and equal in size amongst themselves. The fungus seems to have been observed as a pest of the klippfish only after the 1880 observation. Farlow [4] observed it along with other microorganisms on reddened klippfish and initially described it as *Oidium pulvinatum *later [5,6] when it turned out that this name was already given according to Farlow [7] there is an *Oidium pulvinatum *Berk. U. Curt., while Saccardo [8] only mentions *Oidium pulvinatum *Cooke (in Grevillea) that lives on *Carya tomentosa *in South Carolina, compare also Ravenel [9]), as *Oidium morrhuae*. Saccardo and Berlese [10] recognized the fungus as *Torula *and called it *Torula pulvinata *(this name must remain with *Alysidium pulvinatum *Bonorden [11]), which lives on pine wood. Also, compare with Saccardo [12].

Consequently, the Nordic researchers in particular have been busy with *Torula*. This fungus is particularly prevalent and damaging in the Norwegian operations. Armauer-Hausen [13] (not seen), is already supposed to have shown in 1883, that that, which one called "mites" (mid) on klippfish, was fungal vegetation.

O. Johan-Olsen [14,15], in the year 1886, distinguished true mite, red mites and brown or black mites. In the latter, he recognized a fungus, which he considered new, and named it *Wallemia ichthyophaga *in honor of a Norwegian fisheries inspector F. M. Wallem, who had made attempts to fight the pest and also was persuaded of the fungal nature of the same. It was *Torula epizoa*. Johan-Olsen gives a good description of the fungus. He also grew cultures on artificial nutrients, and he recognized the importance of the conoids for the dissemination of the pest. As counter measures, he recommended cleanliness and disinfection. Later, he is supposed to have found the fungus on salted meat as well (according to Gran [16], see below).

At the same time Brunhorst [17-20] did research on the fungus. He saw the kind of germination of the conoids, which in general, swell and form transverse walls in all directions, but no germination tubes, which he thought was peculiar. Oddly though, he did not consider them particularly necessary, but he thought of it as a consequence of adaptation to the salt levels of the nutrients, and therefore not so essential for placing the fungus in a particular status. He found that the packing houses pose an unusually rich opportunity for infection, even though not the only one, and that the infection of the fish in general occurs while layering or immediately thereafter, not before, for example not already at the drying stations. Experiments that he performed (1889), were supposed to have proven that. He also recommends disinfection of the rooms and apparatus in question. Steam water, the way it could be used, was not effective enough. In contrast, attempts with sulfuric acid were adequately successful. Therefore Brunchorst recommends that the packing rooms preliminarily be cleaned with water (without soda or soap) and disinfected, while still damp, by burning with sulfur (1889). In addition, he stated that boric acid, when added to the salt, hampers the development of the fungus, or (with a 10% addition) completely hinders it. It is however, due to hygiene reasons, not permissible to use this substance even in smaller quantities (compare Kister [21]). The same conditions and also the price speak against the use of sodium benzoic acids.

We can only make references to the partially justified attacks of Brunchorst against Johan-Olsen [22].

We owe the essential recognition of *Torula epizoa*, specifically, its role in the klippfish industry, to Kr. Høye, who is extremely busy with an extensive array of publications concerning the fungus [23-29].

Høye succeeded in growing the *Torula *on different nutrient surfaces. It grows on klippfish, on herring and on salted meat. Klippfish is not the ideal nutrient surface for purposes of pure culture, since it is altered by the sterilization process, into a form that is not optimal for the fungus. A certain salt-content is essential: 10% is ideal, but the fungus still flourishes even in nutrients saturated with salt. Mixtures of gelatin with fish soup and table salt is of little use; 20% salt is supposed to keep the gelatin fluid. A particularly good broth was a flour broth composed of 80 parts wheat flour, 100 parts fish soup and 5-30% table salt, with an optimum at 10%. Such nutrients are good for the production of pure cultures and the proof of *Torula*-germs, especially because they allow the development of the few organisms that love or tolerate a high salt concentration.

The main goal Høye set for himself was to clear up the conditions of the appearance of the fungus in the klippfish industry in order to find a way to fight the pests. After identification of the appropriate nutrients that made it possible to comfortably observe the *Torula*-germination and excluding the general development of the ordinary saprophytes, observations were made on the fungal germination on klippfish from different sources and processed in different ways, furthermore the packing- and storage rooms, the ships, the drying rooms, the air on the ships and in the area around the workrooms, the apparatus and countless probes of the ordinary types of salt. It turned out that the befallen fish itself was the major source of the infection. From it, the germs get into the air in the environment, into the dust of the ships and the storage facilities, to the drying places, onto the apparatus and finally, and most importantly into the salt. The common practice of brushing off the attacked fish strongly contributes to the dissemination of the germs. The drying places are practically free of germs in the winter, however in the fall they are often strongly infected. Findings on the storage room walls showed up to a million germs per square meter. The salt, however, seems to be the main carrier and transferor of the fungus. The production sites are either fungal free or almost fungus-free. But they are infected during shipment in infected ships or in storage at infected storage sites, and the germ contents can rise to 35,000/kg and higher. Also, it seems possible that the germs reproduce in the salt, if organic dust, for example flour dust, falls on the salt in the storage facilities.

The main infection of the fish occurs during the salting. The source and the kind of salt used as well as the difference in the hygroscopy of the kinds do not have any influence. However, the germ content and the conditions in which the initial development of the germs is encouraged or inhibited are critical. The sooner the fungus arrives on the fish, and the further it can develop initially, the stronger the later attack; the first two to three weeks are the critical time. The treatment method, stronger or weaker pressing, the degree of dryness reached, are, according to Høye, in contrast to Gran's observations (see below), without substantive impact. Though strong drying does hamper the development; at a fish water-content of less than 30% the fungus can no longer grow. But one cannot bring the water content down that low, only little fish can get as low as 34%, large ones only 40%, and at a water content of 36-40%, the fungus grows well. Also, low temperature only protects so long as it is there; temporary cooling to -19° is tolerated without damage.

On the basis of these observations, Høye sees disinfection as the only means to fight the evil. All rooms, in which the fish and in particular the salt in which it is sent, layered, or worked on, and all apparatus, in which they come in contact, must be disinfected; the salt should optimally reach the location of use directly from the ship in which it comes. In order to disinfect, the suitable recommendation is, after thorough cleaning and washing the rooms, if they are sealable, to burn sulfur (30 g/1 cubic meter) or wash with formaldehyde containing water (1-2% of 40% solution). Attempts to follow this procedure have, for the most part shown the usefulness of the process; it was successful in to reduce the numbers of germs on the fish from 1/100 and even to 1/500. But the method is difficult to implement.

In addition to *Torula epizoa*, Høye mentions a few other, more or less fungal vegetations, on the klippfish. What he calls Tangsop a and Tangsop b, seems to be identical to *Torula epizoa*. In contrast *Torula pulvinata *seems to be sufficiently different. It is equally salt-loving, thrives on the nutrients containing 30% salt, but is of little importance for the fish and was not further researched.

A bit later than Høye, Gran [30] began research and experimentation to fight the klippfish fungus. Gran considers it out of the question that disinfection of the fungus would be a successful way to fight it, he believes, rather, that the method of treating the fish during processing has an influence on the thriving of the fungus, and hopes, to fight it using this knowledge. With the aid of significant means provided by the Norwegian Fish regulation, he got five large shipments of fish, each consisting of 6000-7000 kg, treated in different ways, and compared slaughtered and not slaughtered, deeply cut and flat cut, washed with salt and not washed, salted in piles and in salted in the lake, firmly pressed and weakly pressed with regard to the appearance of *Torula*. The two shipments, A and E, that later on had the lowest moisture content (37.5-38.3%) and the highest salt content (21.5%), remained fungus-free, or almost fungus-free. The shipment D, with the highest water content (42.6%) and the lowest salt content (18.4%) was partially befallen (havde ikke saa faa fisk befaengte ved sop) (have not so few fish afflicted with fungi). Gran finds the connection between the treatment and fungal development has proven for him that the more carefully the fish is dried and the drier and cooler it is stored, the harder it is for the fungus to grow.

Høye [31] looks at Grans's experiments with sharp criticism. He finds that it lacks an exact basis, in that there were no conclusions made on the fish's exposure to the fungal germs during preparation. This in no way proven result, that well prepared fish last better, was already long known and would not have needed expensive experimentation. With regard to Gran's conclusion regarding shipment D, Høye seemed to be in error, and Gran ([32], compare to Høye [33]) later corrected it.

In the following time, Gran [34] again returned to his experiments and the oddities of the klippfish. He particularly pays attention to the peculiar ability of the fungus, to grow on strong, and even saturated salt solutions and developing an equivalent osmotic pressure in its cells, and bespeaks the conditions for growth and nutrition of the fungus. The fungus grows between 5° and 37°C and best at 25°C. It thrives best in nutrients with 10% table salt, but even grows on fish where the salt is crystallizing out; but a certain degree of moisture of the fish and the air is necessary. Nitrate rich nutrients with higher nitrogen compounds, such as fish soup, egg white, also peptone, are suitable for the nutrition of the fungus; an addition of glycerin or sugar can work well, but is not necessary. Asparagines, ammonia salts or nitrates are unsuitable as nitrate sources. Grans's work also includes a report about the changes that the fish suffers, based on research by Schmidt-Nielsen, as well as a few notes about the bacteria that appear during preparation (compare also to the essay by F. Duge [35], that adds personal observations in addition to a report of those of Grans' work).

From the perspective of the practitioner [36-38] even lately, the opinion is held that for the appearance of the fungi, the treatment of the fish is critical. Earlier, when one prepared the fish, it was better washed and prepared more slowly and carefully, it is said that there was little fungi. Although cleanliness in the workplace is being upheld as being very important, disinfection meets with little affection.

My own research on *Torula epizoa *led to verification of the facts about the fungus made by the Nordic researchers, especially Høye. This is why I have avoided a further thorough investigation. I made the culture on salt-containing agar produced with klippfish-broth. It was noticed that the conoids initially got larger and then divided, converted into cell lumps or packages, occasionally let a few hyphae sprout and finally began to bring forth chains of conoids at the ends of the hyphae. Finally about 1 mm large half spherical piles had developed corresponding to those found on the fish. Only, they stayed much smaller, and growth was very slow. From this one can conclude that on the one hand the chosen agar nutrients with fish broth does not provide the most optimal conditions for development of the fungus, and that on the other hand the high salt content of the nutrient base slows down the growth. Clearly the cells need to use a lot of energy to create turgor pressure in order to combat the osmotic pressure of highly concentrated or saturated salt solutions.

The advice of Høye's to fight the *Torula *can also find use in the German conditions, as long as the German fish industry works by starting out with freshly caught fish. The factory in Geestemünde is already putting into effect disinfection of its factory rooms with good success. Since the salted and dried fish is still imported from abroad, especially from Norway, is brought in, processed and stored here, the common dragging in of the fungus is unavoidable, and the task still remains to try to see if it is somehow possible to make the germs on the befallen fish become incapable of developing. It seems desirable to restart the research which already started to have good results as soon as the import conditions permit.

## II. The red bacteria

### A. Historical overview

The second pest of the klippfish consists of a conspicuous red coloration that is connected with the process of disintegration and the appearance of a penetrating foul odor. The first to occupy himself with this phenomenon was Farlow ([39], also see the notes under calothroxystis [40]). He considers the causative agent to be *Clathrocystis roseo-persicina*, a peach-blossom colored organism that was first found and described along with other red colored microorganisms at the bottom of waters by Cohn [41]. This *Clathrocystis *also occurs, according to Farlow, in storage houses, for example on woody parts, often at the beach, and is dragged in with the salt. The often-used Cadix salt that is weakly reddish colored is supposed to contain *Clathrocystis*. Farlow recommends using Trapani salt and painting the wooden parts with oil paint, so that they can be washed off.

Red colored lower organisms have been described several times. Farlow references the red algae and bacteria that Dunal [42] found in the Salinas of the Mediterranean, where one lets the salt crystallize out, and to those which Ray Lankester [43] has observed on rotting animals under water.

In addition to *Clathrocystis*, Farlow also found a *Sarcina *on the klippfish, but it is not red, but is instead supposed to be colorless. He gives the size of the cells as "5-8 m" (?). He calls them *Sarcina morrhuae*, but later is convinced they correspond to the *Sarcina litoralis *that was, in the meantime, described by Poulsen [44] as found on fouling slime on the beach, and whose cells are supposed to be 2.66 to 3.99 μ long, whereby he relies himself on a comparison by Paulsen, and now he also calls them *Sarcina litoralis *[45].

A few years later (1884) Bertherand ([46], also compare [47]) reports about appearances of symptoms of poisoning that were supposed to have appeared in soldiers who had consumed red klippfish in Algeria, and Mégnin (compare [48]) and describes a "Protomycete" under the name of *Coniothecium Bertherandi *as the causative agent of this red coloration (there is an even older report about poisonings, that the soldiers of the foreign legion in the province of Oran were supposed to have gotten after consuming spoiled klippfish. But the report written does not state that the fish was red. Compare with [49]). They are pale-red one-, two- or four-celled with colonies whose size is given as 6 to 10 mm (?). According to Saccardo and Berlese [50], this *Coniothecium *is supposed to correspond to the *Sarcina litoralis*. The authors point toward the fact that according to Zopf [51,52], the *Sarcina *(I do not see the Sarcina mentioned by Zopf) as well as the *Clathrocystis*, the latter as Zoogleo, are only manifestations of *Beggiatoa roseo-persicina*. Farlow [53] however, holds fast to the idea that the *Sarcina *and the *Clathrocystis*, that occur next to each other are different, and that the *Sarcina *has no relation to *Beggiatoa*; it completely lacks the red color. Patouillard [54] has the same opinion, and has found essentially the same organisms, specifically *Clathrocysits roseo-persicina*, *Sarcina morrhuae *(= *litoralis*) and some not further described bacteria and fungi on klippfish and on salted pork meat turned red. Farlow [55] also observed red salt pork; but he did not consider *Clathrocystis*, but rather a *Bacterium *or a *Bacillus *is the causative agent. Heckel (according to Dantec, see below) on the other hand believes that *Clathrocystis *is the cause for the red coloration of the klippfish. Layet [56] describes "sarcoden" similarly as one- and two- and four-celled organisms, that could well correspond to the *Sarcina*. He believes them to be algae but leaves the question open, as to whether they are "a *Beggiatoa *from the family of Nostocaceen, like Farlow's *Clathrocysits*". Johan-Olsen [14] also studied the red coloration, the supposed "red mites". He finds a *Sarcina*-like organism, *Sarcina rosacea*, with 0.3 to 0.5 μ sized cells, that are supposed to make gelatin become liquid, on solid media, form round colonies, make a *Merisopedia*-like skin on liquids and supposedly make the fish smell foul. Other contemporary observers, Gayon and Carles ([57], according to Le Dantec, see below) have raised a chromogenic bacterium on a salt rich nutrient surface made of red fish.

Further cases of poisonings by fish that had turned red were observed around the same time in St. Petersburg (according to Le Dantec, see below) and in Lorient (1884). In the case of Lorient Berenger-Feraud (according to Layet [56], and the writings by Bérenger-Feraud [58], was not accessible to me), he considers a fungus to be the cause of the red color. The question of the cause of the poisoning is answered by Layet (see also Roumeguère [59]) and most of the other judges, as follows: the red fish and its organisms as such, are not poisonous, but rather, that through a foul degradation, alkaloids (ptomaines) are formed occasionally with or without the red coloration.

The opinion that the red coloration is caused by a bacillus soon appeared from Edington [60] and as a result of this research, also from Ewart [61]. In cuts through red fish flesh, only micrococci were found, and they were more or less in deeply invading gaps and at intramuscular septa. Of eight different bacteria (*Bacterium*, *Bacillus *and *Micrococcus*) that Edington isolated according to pure culture methods, none showed a red color. Contrary to this, a red growth was obtained when parts of the red fish were transferred to bread-dough (bread-paste). By fractionation and dilution, a bacillus was isolated, which forms 1.5 to 4 μ long and 0.3 to 0.5 μ thick rods, occasionally Leptothrix-like threads which can be 25 μ long and it brings forth spores both in the rods in the threads. It gets the name *Bacillus rubescens*. This organism grows poorly on Koch's jelly (gelatin) and on agar at normal temperatures; at higher temperatures, if the gelatin remains liquid, it makes a skin on the surface that colors weakly pink on the gelatin underneath, while on agar, it remains grey and, like oil paint that was left out in the sun, it turns rippy and gets bubbly. On bread dough the clear red color appears: "If merely moist, a red or eosin-pink color will develop in a few days at the inoculated area, and this spreads slowly to the periphery. As it gets older, the red coloration passes down the sides of the flask and there becomes of a deeper tint." Edington believes that this bacillus is the cause of the red coloration of the fish. He also isolated it out of the salt with which the fish was salted; he does not, however, give further information about where he got the salt. Subcutaneous inoculation of guinea pigs remained without any pathological consequence. Boric acid (3%) hampered the development of the bacteria.

Whether the nutrients used by Eddington were high in salt or not was not stated by the report.

An in-depth study was soon performed by Le Dantec [62] (1891). He distinguishes between two grades of red coloration. Fish befallen in the first grade has a lightly removable, non-sticky film cover, under which the meat is still firm and healthy. Therein a weakly greenish alga was found, that Le Dantec considers, probably to be *Clathrocysits*, which corresponds to the Protomycetes or the "Algae" of the earlier observers, furthermore bacilli and cocci were found. After two to three months, the first grade converts to the second grade. Then the fish is covered by a sticky red mass, it shows an alkali reaction and gives off a nauseous smell. Now a *Sarcina*-like *Coccus *whose cells are often united to groups of four appears. The seeding onto artificial media gave only common bacteria, on a forgotten dish, however, a red colony was found on top of a white one. The white colony was composed of small cocci, the red one contained mobile 4 to 10, even up to 12 μ and more long rods with spores at one end. Pure cultures could be obtained by removing the areas where there had been no growth from the forgotten bowl, and mixing them up with new gelatin and thus forming new plates and then, after eight days red colonies were obtained. Furthermore the resistance of the spores to warmth was used: the diluted red mass was warmed in a thinned out capillary for a minute, to 95°, and then seeded out. The cultures gave more red coloration at 10 to 15° than at higher temperature. In liquid nutrients (bullion) there was only turbidity, no coloration. There are no details given about the composition of the nutrients, specifically about their salt content, there is only mention of bouillon and gelatin. The fish was colored red, but fresh fish was better than sterilized ones. Le Dantec calls the organism "red *Bacillus *of Newfoundland" (bacille rouge de Terre neuve). Krause [63] later calls it *Bacillus Danteci*.

Furthermore a *Coccus *was isolated, that received the connotation "coccus of the cod red coloration" (*Coccus *du rouge de morue). It appears red colored in both stages, most apparently in the second stage. It grows poorly on artificial nutrients, the above described method was unsuccessful, and one only obtains it accidentally if one starts numerous bowls. It lives on the fish together with other microbes, notably with a small, liquefying *Coccus*. Together with this, it produces a lot of red color, while when alone, it does not give the fish a color. The diameter was taken to be 3 to 5 μ.

Besides these two organisms, a pink yeast was found, along with a reddish mold and several cocci that bring about a yellow color. The red-colored fish turned out to be harmless when consumed. It can, however, become dangerous if it is badly decomposed. Le Dantec has no specific judgment about the cause. He does, however, make the storage rooms and apparatus responsible too, and he declares the increase in frequency a result of longer storage. As a counter-measure he suggests the use of preservatives at the stage of salting.

Later on Le Dantec [64] returns to the matter. He did not find the red bacillus again, but out of the 25 probes he kept getting another, very unusual bacillus, that he now considers to be the cause of the red coloration. This makes 2-15 μ long, sometimes almost threadlike rods without spores. It is immobile in the red masses on the fish, but is easily motile in saltwater. It is Gram-negative. At 68-70° it is killed within a minute. It only grows on nutrients over-saturated with salt. Sodium is specific to it and cannot be replaced by potassium, calcium or magnesium. In a bouillon made of 20 g fish, 80 g salt and 200 g water it appears and develops at once (d'emblee) and can be isolated out of it on solid nutrients. The bouillon is covered with a red veil after 5 to 10 days. By the way, it grows very poorly on the artificial nutrients (miserablement), and its original base is the salted fish. Here it is accompanied by other bacteria that seem to enhance its development, a *Sarcina*, a Gram-negative *Coccobacillus *and a long Gram-positive *Bacillus*. Successful isolation of the red *Bacillus *from the sea salt from the Mediterranean and from Lisbon was possible. Le Dantec recommends, therefore, that the salt used for salting, should be pasteurized.

Høye (loco citato [65]) also addresses the red colorization of the klippfish. He points out that Torula mainly occurs in Norway, while the red colorization occurs more frequently in the industries of France, Iceland and the Farao Islands. In his publication in 1904, Høye mentions a red *Sarcina *and a white *Sarcinomyces*. The latter is a bit dangerous, although it often occurs in such large quantities that the white granulated cell piles almost cover the whole fish. The red *Sarcina *on the other hand, is said to make the fish foul-smelling and unusable. In his 1906 work, Høye names a number of other salt loving or salt tolerant organisms in addition to *Torula epizoa *and *T. minuta*: *Sarcinomyces islandicus*, presumably corresponding to the just mentioned *Sarcinomyces*, with cells from 6 to 7.7 μ in diameter, that are joined to two, four or more cell-packages, that it occurs especially on fish from Iceland, is however without much importance. This applies even to a higher degree for two other kinds, *S. niger *and *S. sporigenus *(the two types of sarcinomycetes described by Linder [66] forms two types: *S. crustaceus *and *S. albus *which both form short, poorly developed threads, whose cells divide to form sarcina-like packages. *S. crustaceus *forms sprouting conoids. More detailed studies are missing. *Sarcinomyces islandicus *is mentioned by Høye without authorship and literally without references; seemingly he gave the name himself. Compare also Lindner, 4. Aufl., 1905, 324 and Lindau, Pilze VIII, 10 in Rabenhorst, Kryptogramenflora).

*Micrococcus α*, with cells with a diameter of 1.2 to 1.3 μ that often take on the form of diplococci, makes reddish yellow colonies. *Micrococcus β*, with cells of 1.2 μ in diameter forms waxen yellow colonies. *Bacillus γ *makes pale colonies with very motile rods from 2.5 to 4 μ in length and 1 μ thickness. All three bacterial types grow best on nutrients with 10 to 15% table salt content. They do however have no connection with the red coloration of the fish and are basically unimportant for the fish.

Apart from these organisms, Høye also found three yeast types and a few mycelia, namely *Hormodendron halophilum*, *Penicillium glaucum *and *Aspergillus glaucus*. The red *Sarcina *of 1904 which had been said to be the causative agent is not mentioned again.

In his 1908 treatise, Høye, in addition to the *Micrococcus β *and the *Bacillus γ*, also mentions "red Bacteria", that particularly occur in the salt storage. They grow only on the surface, not into the fish, and they consist of somewhat oval cocci embedded in jelly and often united into diplococci; their size is about 1 μ. Their growth is very slow, both on flour as well as porridge.

Lately Beckwith [67,68] describes what he believes is a new halophytic diplococcus, which he found, along with *Calothryx*, *Sarcina *and different other bacteria on red fish. He calls it *Diplococcus gadidarum*. The cells are 0.4 to 0.5 μ large and Gram-positive. Two years later 1 μ involution forms developed. In addition to the culture bullion, fish broth or fish drippings with 2% agar and 5 to 10% salt was used for the culture. This table salt content is supposed to give the best conditions. On these types of nutrient surfaces, salmon-colored colonies with a 1-2 μ diameter occur within 96 hours at 20 to 30°C, which, after repeated inoculations, become paler. In smear specimens of fish flesh the diplococcus is the dominant bacterial form. Beckwith considers it to be the cause for the red coloration and considers it to have the most degradative property, but does admit, however, that the dominant bacteria perhaps change.

According to Kellerman's [69] research, Høyer's *Microcooccus *(1908) and Beckwiths's *Diplococcus gadidarum*, correspond to the *Sarcina morrhuae *or *S. litoralis *previously described by earlier researchers. Beckwith's *Diplococcus *is not supposed to be a homogenous kind, but rather consists of two different kinds, that Kellerman describes and distinguishes as *Micrococcus litoralis *and *Micrococcus litoralis gadidarum*.

*Micrococcus litoralis *lives alone or in cell groups, forming salmon-colored colonies of 1-2 mm in diameter; the cells are bigger: 1.2-1.6 μ. *Sarcina litoralis *Poulsen, *Clathrocystis roseo-persicina *Farlow and *Diplococcus gadidarum *Beckwith (partly) are supposed to be synonymous.

*Micrococcus litoralis gadidarum *mostly lives alone, seldom in groups and it creates brilliant vermillion colonies of 0.5 to 2 mm in size; the cells are smaller, 0.35 to 0.5 μ. *Diplococcus gadidarum *Beckwith (partly) is supposed to be synonymous.

The larger kind is more resistant to drying and generally grows faster; and in concentrations above 20% table salt the smaller kind grows better, but both can tolerate 30%. They grow particularly well when grown together. Both are Gram-positive, they do not liquefy gelatin.

In addition to the two mentioned kinds, the following were found: a smaller, orange colored, a yellow, and a colorless *Micrococcus *and furthermore a bacterium of 2-4: 1 μ size and a small, motile organism that was probably a protozoan.

Kellerman considers it to be probable that the bacteria that color the klippfish live in the salt marshes of the coast, and that they are able to tolerate extended periods of dryness. He points to the fact the organisms that Peirce isolated from concentrated salted sole from the coasts of San Francisco Bay, where they produce sea salt, are probably identical to the klippfish bacteria. It is not clear whether Kellerman's opinion is accurate.

Peirce [70] denotes his organisms that belong to the fouling bacteria of the saltwater as "Bacilli", but only gives their measurements as 3.4-3.6; 3.2-3.3 μ, which by no means are the measurements of bacilli, but rather point towards coccal forms, but seem unusually large even for cocci, and considerably larger than the measurements given for Kellerman's micrococci. Incidentally, those "bacilli" are also supposed to be living on the crystallizing salt and to color the latter and the sole red. The possibility appears, and Pierce also considers it, that they arrive at the klippfish via the sea salt and participate in its reddening. The fact that experimentations by Peirce were able to cause the vegetative reddening of the klippfish through the transfer of the bacteria does, however, not actually prove that the bacteria caused the klippfish reddening. The report that it was possible to gain the same red colonies from the reddened klippfish is of more significance; however, it loses worth, by the fact that Peirce does not say anything about a comparison of the two probable sources of the cultured bacteria and that there are no illustrations. A limitation to images of culturing glasses with cotton stoppers in preference over pictures or the red bacteria themselves would have been much more preferable. It might also be noticed that the red color that was supposed to change from "pink" via "clear red" to "crimson" is supposed to come out of the colonies as they differentiate and are supposed to color the agar pale red.

At the end one should also point toward some papers mentioned in the literature that I was unable to obtain hitherto ([71-73], a coccus was supposed to have been described here, that was quite different from Beckwith's *Diplococcus*).

### B. The bacteria found by my own research

My own observations partially depend on the experiences that I had when I repeatedly visited the klippfish storage facility in Hamburg Harbor and the klippfish facility at Oxstedt near Cuxhaven, but for the most as a result of the research on the countless probes I took along with me or I was sent to obtain. The red coloration appears mainly during the Summer and Fall months but even in Winter it can develop, once it is present. A particularly strong attack was observed in the Fall of 1917 in the storage facility at Oxstedt and in Cuxhaven. Strongly affected fish can already be attracting attention from a distance due to its reddish color. The red coloration is apparent superficially on the surface of the skin as well as on the flesh side uncovered by the preparation; it can also invade deeper into the flesh via the gaps that form between the muscle bundles. One finds red drops, red smeary covers or also a seemingly diffuse drenching of the fish flesh. The color tone is close to vermillion or somewhat paler, often with a touch of orange-red.

#### 1. The red bacillus

##### a) The cultures

Since from the start, it appeared as though it was a bacterial attack, the experiments were directed at the isolation of red bacteria. Seeding on unusual bacterial nutrients did indeed always give numerous kinds of bacterial colonies, but mostly whitish, yellowish, light-brownish or grayish in color, only seldom strongly yellow or reddish colored ones. In contrast it was easy, through the transfer of tiny amounts of red fish flesh or even by starting through needle pricks from red fish flesh to cause red coloration on pieces of healthy klippfish that gradually spread from the inoculated parts of the fish. Since these klippfish pieces had to be kept moist and had to be protected from common fouling agents, they were laid in sterile Petri dishes on a moist layer of common salt, which also had been sterilized right before. Thereby it often became apparent that the salt, that gradually got soaked with the substances released from the fish, gradually took on a clear pale red appearance. This corresponds to the observations in practice. Red colored salt has often been noticed in factories and storage facilities and was already apparent to earlier observers as was repeatedly observed above, and led to the question that if perhaps the red coloration came from the salt and if the red organism originally lived in the salt.

After these experiences, one could presume that the sought after bacteria have a definitive preference for salt. Therefore, strongly salt-containing nutrient bases were made, at first made of agar with a decoction of the klippfish and enough salt addition up to saturation. But even on these nutrients, the desired result was not gotten at once.

The presumption, that perhaps, through the heating, there were resulting changes in the nutrient base, that affected the growth of the bacteria in an unfavorable way, led to the decision of doing experiments of growth on uncooked, germfree, nutrient base seem desirable. Such nutrient bases were obtained in different ways:

1. Smooth-cut small klippfish pieces were covered on the previously sufficiently dried surface by a colloidal layer. They were then laid in sterile Petri dishes on damp common salt and inoculated on the colloidal layer. It is presumed that the colloidal layer separated from the ethereal solution does not contain living germs, and that a germ-free fish extract diffuses through the colloidal layer.

2. Thin tiles of fired and porous clay or thin plaster plates that were previously sterilized were put on moist small pieces of klippfish which lay on common salt in Petri dishes. After the plates were soaked through with the moisture from the fish they were inoculated on the surface. Here too, one could presume that the liquid that diffused through the plates were germ free.

3. Through the pouring over of macerated klippfish with a bit of water and two- or three-days of soaking, a solution was made, consisting of the soluble material from the klippfish. The solution was saturated with common salt, cleared by filtration, and then made germ-free by filtering through a Berkfeld filter (The filtration through the Berkenfeld filter was enabled by Herr Dr. Kammann at the Hygienic Institute in Hamburg with the available resources there). In order to make cultures, the following components were used:

a) sterile common salt, that was soaked with this solution,

b) sterile clay tiles, that were laid on the soaked common salt,

c) common salt-saturated three percent agar that, after sufficient cooling, before solidifying, was quickly mixed with an approximately equal amount of the solution.

As already mentioned, there was a sometimes remarkable development of red coloration in the common salt that was soaked with the solution of fluids diffusing out of the red fish. The same coloration could be created artificially, if some of the red mass or some reddened fish flesh was brought into common salt, which was soaked with the germ-free fish extract.

One succeeded in obtaining red bacteria colonies by inoculating with the liquid of such common salt as well as by rubbing the red mass removed directly from the fish. It was possible to obtain the red bacterial colonies on the clay tiles and the colloidally covered fish flesh. The development, however, went forth very slowly. The cultures on the clay tiles appeared as the cleanest and most convenient and were therefore used preferentially later.

The obtained colonies developed clear-looking drops with a lively vermillion red color that slowly spread out in a fatty, gleaming layer over the clay tile or the colloidal skin on the fish flesh. They were almost entirely pure from the start or became pure through transferring once or twice, till pure, since most of the other bacteria either grew poorly or not at all on the strongly salt-containing nutrient surface. The transfer onto fish flesh also brought forth this strong red coloration on the fish flesh as well.

The following is a short overview of the development of a few culture sequences:

##### I

115. 9. Sept. 1916. Transfer of red fish in a bowl of salt that was soaked with fish extract. The salt colors pink.

176. 17. Dec. 1916. Transfer from salt bowl 115 onto agar with fish extract. Next to white and yellowish colonies there are strongly red colored ones on 23. Febr. 1917.

253. 28. Febr. 1917. Transfer from a red colony from bowl 176 on a clay tile that is lying on fish. On 26. March, weak, on 17. June, strongly red coloration on the tile.

296. 29. March 1917. Transfer from 253 onto fish. Clear red coloration on 30. April, strong on 17. June.

##### II

144. 17. Dec. 1916. Transfer from salt bowl 115 onto a plaster plate that is lying on fish. On 19. January 1917 red colonies between whitish and grey.

183. 24. January 1917. Transfer from 144 onto agar with uncooked fish extract. On 23. Febr. Red colonies.

276. 28. Febr. 1917. Transfer from 183 onto the same agar. Individual red colonies on 26. March.

##### III

154. 17. Dec. 1916. Transfer of red fish onto a clay tile that lies on a small fish piece. On 2. Jan. 1917 clear red drops have formed.

156. 2. Jan. 1917. Transfer from 154 onto fish that is covered with colloidal skin. On 20. Jan. clear red drops are present.

201. 27. Jan. 1917. Transfer from 156 onto a clay tile that lies on common salt with germfree fish extract. On 10. Febr. Red drops have developed.

236. 13. Febr. 1917. Transfer from 201 onto agar with germfree uncooked fish extract. On 12. March red colonies. On 26. March colonies have paled.

285. 26. March 1917. Transfer from 236 to the same agar. On 16. April red colonies. On 17. June the colonies have paled.

284. 26. March 1917. Parallel examination to 285 onto agar with cooked fish extract. On 16. April red colonies.

##### IV

179. 21. Dec. 1916. Transfer from salt bowl 115 onto a fish piece covered with colloidal material. On 29. Jan. red colonies present.

**Figure 1:**
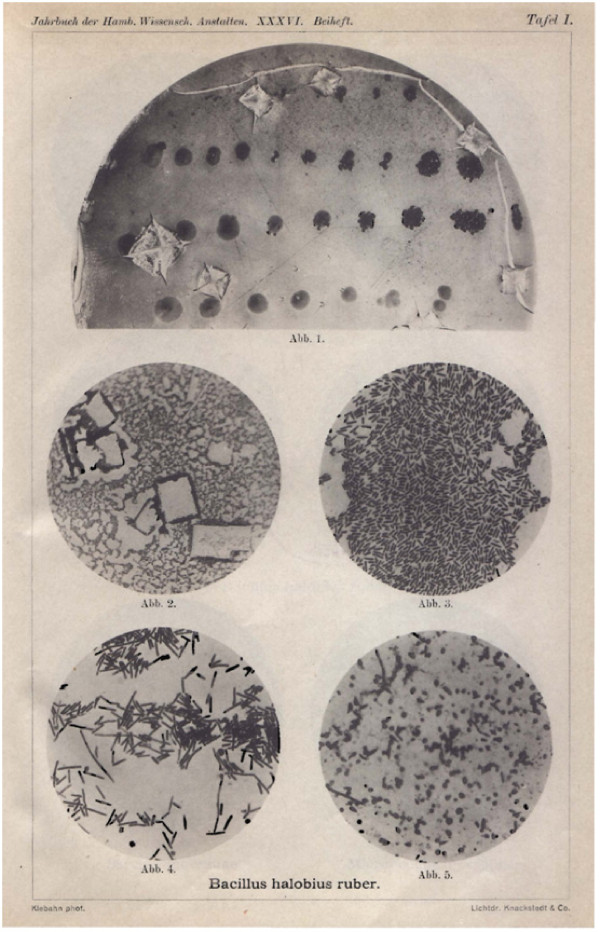
**Images 1 to 5. *Bacillus halobius ruber***. **Image 1 - **Part of a culture on fish agar in a Petri dish. Inoculation of the tile through needle stabbing in rows. On one part of the resulting colonies of yellowish-grey bacteria, red colonies of *Bacillus halobius ruber *have developed that in the photograph appear black and are noticeable due to their odd gleam. The saturation of the culture with common salt appears at several places with the crystallizations of the salt, and the age of the culture at the same time with the rips that have arisen at the edges of the crystals and near the border of the dish as a consequence of the drying agar. Enlargement 3/2. **Image 2 **- Bacteria smeared out in saturated common salt solution out of the culture on a clay tile. The light quadrants and squares are the places where crystals had been formed. Enlargement 370/1. **Image 3 - **Bacilli of a similarly made preparation. Enlargement 750/1. **Image 4 - **Little rods and longer threads. Smear out of a culture in liquid nutrient base. Enlargement 750/1. **Image 5 - **Altered little rods out of an old culture on horse serum with saturated salt solution smeared out. Enlargement 750/1.

181. 24. Jan. 1917. Transfer via needle pricks onto fish agar. On 5. Febr. Yellowish colonies that spread out into circular colonies. From 12. March onwards red colonies appear on the pale ones that gradually enlarge and multiply in numbers. They partially flow together, forming oddly branched figures, separate sharply from the pale bacteria, on which they apparently flourish excellently. In the photographic record of 18. April (Figure 1, Image 1) the red colonies contrast sharply with the pale bacteria by their dark color and their odd shine. At the same time the common salt crystallizations that develop at saturation of the nutrient base are apparent in the image. The bowl could then, by gradually drying, be preserved for longer time.

After success in obtaining red colonies, they could be grown on different nutrient bases. In the beginning, it seemed as if the fish extract obtained by cooking was a bit less of an optimal nutrient base, but later on red colonies were obtained from agar mixed with cooked fish extract when the transfer was made out of such colonies. Besides the above mentioned case (bowl 284) I have therefore, the new ones aside, no less than ten older parallel cultures as examples, of which two go back to salt bowl 115 and eight go back to the clay tile cultures. If on agar nutrient bases, no matter which kind, numerous colonies develop next to one another, so densely that they flow together and that the surface of the agar appears as though it is covered with a shiny, pale red to deep red colored lacquer layer. The shine and the clear appearance in general belong to the particularly significant features of the colonies of these red bacteria that are detected by the naked eye. In addition it should be pointed to the preference of these organisms for high salt content and is not unusually recognizable by the fact that red colonies sit directly on the crystals that extrude from the nutrient base.

The Hygienic Institute prepared horse serum based nutrient base, which was recommended to me as a very advantageous nutrient base for some cultures and for isolating germs by streaking of the appropriate nutrient base, and it was prepared for my purposes, saturated with salt and supplements of peptone or peptone and glucose (each at 1%). The following facts turned out: on the surface, which was rather firm, the germs could indeed be separated well by rubbing, the cultures which developed were strongly colored, and the bowls could be preserved for months if mould fungi, not all of which were disturbed by the salt content, did not accidentally invade. I was able to preserve a few tiles up to nine months. The red colored colonies stand out advantageously from the light, yellowish-white color of the serum. In older tiles however, they start to pale and take on a violet-brown tone.

Another advantageous nutrient base is a mush of corn meal with an addition of fish flesh extract. I was led to test this nutrient base on the one hand, by the indications by Høyes that the not seldom occurring contamination of the salt with flour dust favors the multiplication of the germs of the klippfish damaging agents, on the one hand, and on the other hand, the cultures of the *Torula epizoa *on flour mush. Since I had some corn meal at hand, I used this for the experiments. It turned out that on this nutrient, these bacteria did grow, however, only well, when, in addition to common salt, they received cooked or uncooked fish extract. Also here, the fatty, gleaming, luminescent red covering of the bacterial colonies granted a conspicuous sight. The corn mush soaked with the cooked fish extract sometimes gave a stronger red coloration than the one soaked in uncooked extract.

Cultures in a liquid nutrient base, for example in fish decoction or in uncooked fish extract made germ-free (saturated with salt of course) did not lead to significant development. It develops a whitish deposit and, only in the middle, where it is most dense, a pale reddish one. The liquid experiences a slight turbidity, especially on the surface. The relatively poor prospering of the bacteria in the liquid could have to do with the fact that they have a high need for oxygen. This showed itself even on the clay tiles and salt-bowl cultures. When in such a culture, the clay tile was covered with a red layer, and the common salt was colored pink, the red color was absent under the clay tile.

With varying success, further experiments were carried out on a number of further nutrient bases. It turned out that the red organism is very sensitive to the kind of nutrient bases and therefore can only develop on a limited number of them.

In the following I present a list of the nutrient bases used together with details about the development of the bacteria. All nutrient bases were saturated with common salt.

1. Clay tiles on unsterilized fish pieces or on common salt that was soaked with germ free fish extract or with fish decoction: development very good.

2. Common salt soaked with germ free fish extract or with fish decoction: liquid pink to red, long rods or strings.

3. Fish extract or fish decoction, saturated with common salt, but without further additions: weakly colored sediment, liquid a bit turbid.

4. Agar nutrient bases: a) with fish extract or decoction: good; b) with Leibig's flesh extract: quite good; c) with peptone, asparagine and fructose: very weak; d) with slop extract (compare Klebahn [74] for a very suitable nutritional base for many fungi): no development; e) with albumin: no development; f) with fructose: no development; g) with yeast extract (used at the Hygienic Institute for bacterial cultures as a substitute for meat extract), with and without peptone: no development.

5. Albumin, dissolved in water and then coagulated by heating: no development.

6. Gelatin with fish extract: quite good.

7. Horse serum with 1% peptone and 1% fructose or with peptone alone: very good.

8. Flour mush (made of corn mush): a) without addition: development weak, but color strong; b) with fish extract or with fish decoction: very good; c) with Liebig's flesh extract: good.

Cultures at different temperatures provided some information about the warmth requirements of the red organism. I could use two thermostats from the Hygienic Institute that are continuously set at 22° and 37°, as well as a thermostat of the Botanical Institute, that was set at 29°. It turned out that the bacteria grew better at 29° than at 22° and occasionally at 37° apparently even better than at 29°. A convenient solution to the task of finding out the temperature requirements more accurately would have made necessary the setting up of a greater number of thermostats. The essential difficulties arise from the slow development of the bacteria. What is more, at temperatures above 37° this slow development makes the nutrient bases dry out too quickly.

The reaction of the cultures was neutral or very weakly alkaline. A bit more alkaline reaction, but only in trace amounts, was shown by the reddish colored fluid caused by the bacteria in the Petri dishes, in which I had brought on the development of cultures on klippfish pieces that were lying on salt. Meanwhile this liquid probably contained other bacteria apart from the red ones.

##### b) Microscopic examination

**Figure 2:**
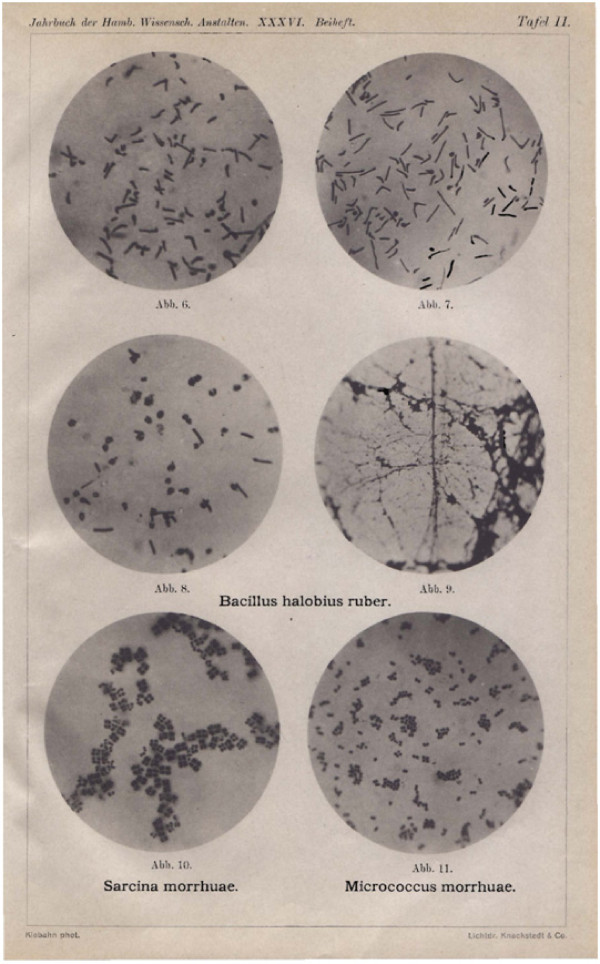
**Images 6 to 9. *Bacillus halobius ruber***. **Images 6 and 7 - **Changes of the little rods under the influence of water or diluted salt solution, starting conditions: swelling of the little rods at the end or in the middle. Bending, rounding. Enlargement 750/1. **Image 8 - **Through the influence of water or diluted salt solution strongly altered little rods: swelling to spheres, to which sometimes still unchanged remainders of little rods are clinging; more or less dissolving spheres. Enlargement 750/1. **Image 9 - **View of a preparation obtained by smearing in pure water. The jelly caused by the dissolution of the little rods has shrunk to a more or less densely net-thready mass, in which there are fine grains or droplets to be found. Enlargement 510/1. **Image 10 - ***Sarcina morrhuae*. Enlargement 750/1. **Image 11 - ***Micrococcus morrhuae*. Enlargement 750/1.

It was noticeable that at first all attempts to identify the bacteria with colored microscopic preparations were unsuccessful. If one uses the point of a platinum wire to bring a bit of the colony into a drop of water and rubs it around in order to distribute the bacteria, there results a strong swelling, threadlike jelly, that only with effort can be somewhat evenly distributed and whose quantity in proportion to the probe used is conspicuously large. If after drying one colors in the usual fashion, one finds, upon examination, a threadlike and netlike coagulated mass, in which more or less clear points of very small size are distributed (Figure 2, Image 9). For a long time, I was of the opinion that they were extremely small coccoidal bacteria that were only present in small numbers and were distinguished by their very strong formation of jelly. Not until the examination of uncolored and untreated cultures crushed between cover glass and slide carrier - without addition of water lead to the right way of recognizing that the red mass was composed of rather big densely crammed together little rods. If one adds water, then, in the area surrounding the bacteria, there is a surgence of a mass of lively streams in the area of the bacteria, which make exact observations impossible; but one obtains the impression that the bacteria bloat and become puffy, and swell up. They seem to have to adapted so much to the saturation of their nutrients with salt or to have gotten accustomed to the saturation as a result of the culturing that, upon addition of water, they immediately explode in a way as a result of the stopping of the external osmotic pressure and are converted into threadlike slime. The process reminds one of the behavior of certain deep sea creatures that burst due to their internal pressure, when they are taken out of the deep.

After this completely understandable, but at first unexpected fact was determined, it was also easy to color the bacteria. The following method usually gives good preparations. One rubs a specimen taken by the point of a wire out of a colony in a small droplet of concentrated salt solution on the slide (the droplet must be as small as possible). One lets it start to dry, kills it with a drop of diluted iodine solution, washes it out with alcohol, after drying heats it in a flame, washes it out with water in order to remove the common salt and then colors it for example with methyl violet 5 B. The immediate spreading of the culture is unsuitable, because the rapidly drying mass spreads too thick and too uneven; a bit better, also only with less than satisfactory results is delivered by the spreading with a sterile brush. First of all in certain areas in the preparations one notices where the common salt crystals had been formed; they appear as free quadratic areas within the more or less evenly distributed bacteria among them or the bacteria gathered around the former crystals (Figure 1, Image 2).

These themselves are recognizable as little rods. Their thickness is 0.5 to 0.8 μ. Their length is variable and dependent upon the composition of the nutrient base. In the red drops, which keep them densely packed, they are relatively short, mostly 2 to 5, but occasionally up to 10 μ long (Figure 1, Image 3). In the liquid nutrient bases, especially in the liquid in the salt bowls mentioned above, they partly form long threads. Lengths up to 45 μ were measured (Figure 1, Image 4). In older cultures, particularly on horse serum, they change their appearance and appear as irregular, roundish, oval or even polyhedral, partly not longer than thick, and almost like cocci (Figure 1, Image 5). These formations might correspond to those indicated as involution forms, but one is not able to know their essence. Lengths of 1.7 to 2.7 and thicknesses of 1 to 1.5 μ were measured. The red color, still clearly recognizable also under the microscope as the pale reddish sheen in greater accumulations of the bacteria, is not to be found at the released individual cells in the salt solution. The color must be contained in the cells, since it does not diffuse out of the living cells, as the observation of the cultures on agar or serum has taught us. The little rods are distinctly negative in Gram-staining. They were colored on the same slide carrier at the same time with clearly Gram-positive *Staphylococci*. More under *Sarcina*.

The bacilli found in liquid environments, for example those growing in the above mentioned salt bowls or those transferred from the cultures on clay tiles in saturated common salt show an oscillating movement that is stronger than expected if it were solely due to a molecular movement. A noticeable locomotion is, however, barely noticeable. It was therefore desirable to find out if flagella were present. I struck out motile bacteria from a hay infusion along with the bacteria of the klippfish distributed in the saturated common salt solution, quickly beside one another on the same cover slip, killed them - still wet - using osmium fumes, treated them after slightly drying with a weak alcohol-iodine solution, washed with alcohol, after drying drew them - in the ordinary manner - through the flame, then washed out the common salt by distilled water and let follow the flagella stain that essentially was carried out in the method of Zettenow (compare [75]). Herr Prof. Dr. H. C. Plaut from the Institute of Fungal Research at the Eppendorf Hospital was so kind as to show me the method and also to carry out the coloring himself. We came to the unified conclusion that there are no flagella, since they were completely absent also in such preparations where the motile hay bacteria clearly showed them. The movement can therefore probably only depend on molecular movement (Note: In addition to the motile bacteria in the hay infusion, there were also small flagellates (a Monas- type), whose flagella consisted of a relatively very thick, strongly colorable main thread and on either side, seated short, and delicate side branches, that are arranged somewhat like a feathered-like arrangement like on a feather. I have not yet had this time to pursue further the noticeable occurrence. As I have noticed, in the meantime, A. Fischer [76] has already described similar structures).

Regarding the pendular movement of the little rods, it is noticeable that their respectively upper end seems to carry a shiny corpuscle. Horizontally lying little rods or threads and colored preparations, however, do not show special structures. They can therefore not be spores. I rather regard the appearance as a result of the coherence of the light beams due to a total reflection of the light in the optically denser little rods. This coherence of the light is observable in a similar way at the borders of glass sheets or at the end of glass rods. It also explains the glossy appearance of bast fibers or of the cell walls of collenchyma in not entirely thin transverse cuts through plant material.

The changes provoked in the bacteria by the water, is verifiable in the colored preparations, too. If one breathes strongly on a preparation after beginning to dry the smeared bacteria and only afterwards treats it further in the above described manner with iodine and colors it, one sees that the bacteria have changed to a crumbly mass. The crystallizations of the common salt altered by the humidity, too, appear now as star-shaped spreads in this mass. The difference is already apparent at weaker magnification. If on breathing one covers half of the preparation with a cover slip, one succeeds in visualizing in the same preparation both altered and unaltered bacteria at the same time. On one side of the border zone one finds the unchanged bacteria sharply delineated and powerfully colored surrounding the free quadrants where the crystals had laid, surrounding, on the other side the equally strongly colored, but completely formless crumbly mass of the decomposed cells, the complete appearance of which is determined by the new crystallization of the salt. In the border area the bacteria appear pale colored, mostly a bit enlarged, blurry and becoming more and more unclear.

It is, however, not recognizable by this method, in which way the change and destruction of the bacteria occurs in detail. One succeeded in stating this only after a series of unsuccessful experiments. Cultures in common salt that were soaked in fish extract or fish decoction turned out to be the most suitable for this purpose. It was already pointed out that the little rods in these cultures reach a great length. If one takes a drop of the bacteria containing liquid and places it between a cover slip and an object slide and carefully allows a bit of clean water to enter from the edge, one can follow the changes at the border layer between the water and the salt-containing solution. Enlarged by the microscope, the rapid streaming, however, disturbs the observation very much. Under favorable circumstances, however, one succeeds in observing the succession of conditions of a single cell. It is more comfortable to work with permanent preparations. One spreads out the bacteria-containing liquid in a very thin layer on the object slide without a cover slip allowing a trace of pure water to enter from the edge and then one lets the preparation dry. Further treatment and coloring is done in the above mentioned way. In preparations of this type one finds the different transition stages next to one another in the border zone between the unaltered bacteria on one side and the completely destroyed ones on the other side.

**Figure 3:**
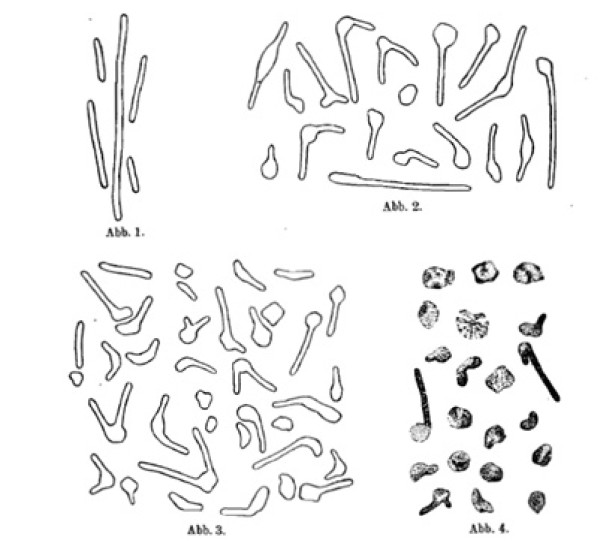
**The red *Bacillus *and its swelling in pure water**. **Image 1 - **Unchanged little rods of different lengths. **Images 2 and 3 - **Beginning conditions of the changes. **Image 4 - **Final conditions; little rods almost completely swollen. **Images 1, 3, 4 - **With drawing apparatus using colored preparations. **Image 2 - **freehand from living material. Enlargement 2700/1.

The reproduced images were obtained in the described manner. The one in Figure 3 Image 2 contains freehand representations of the changes according to living images, the ones in Images 1, 3 and 4 designed by means of a drawing apparatus, contain drawings according to colored preparations. The micrographs (Figure 2, Images 6, 7 and 8) show the juxtaposed occurrence of differently advanced conditions at the same place of a preparation. All stages up to the almost complete dissolving are represented. Compare the explanation of the images.

The result of the studies can be summed up in the following way:

Upon transfer into the solution of lower salt content, there starts a swelling of the cell at some place of the membrane, which must be addressed as the location of least resistibility. Due to the tininess of the object, it is not recognizable whether the swelling of the cell is caused by stretching of the membrane or by bursting and ripping of the same. The place where the swelling occurs is especially often located at one end so that the cells take on a nail- or pin-like shape. Not seldom, however, it is also located to the middle and in this case one-sided, which causes a more or less angularly twisting of the little rod at the affected location; thereby the swollen part is obviously located on the outer side of the angular point crest (Figure 3, Images 2 and 3 and Figure 2, Images 6 and 7). The degree of the influence that depends on the concentration of the surrounding liquid leads either to the remaining of the changes or to causing the whole cells to convert to more or less round tiny spheres (Figure 3, Image 4 and Figure 2, Image 8).

As this representation clearly shows, the change occurs immediately or within seconds after the adding of water. Now the following is clear: The little dried rods, which simply by breathing on them, were dampened, must show different appearance compared to those altered in the nutrient liquid and that the breathed up on preparations therefore cannot give any clarification about the occurring changes.

The sensitivity of these bacteria to alterations of the osmotic pressure and the devastation they experience upon the appearance of water explain why it was only possible to obtain cultures of the red bacilli after lengthy unsuccessful attempts and only by coincidence. If upon dilution one uses liquids, the osmotic pressure of which cannot be borne, the bacilli die and the inoculation is unsuccessful, or only such randomly existing impurities develop that are adapted to the lower salt content or are less sensitive. The great sensitivity also probably explains why there are still nearly always some individual little rods in the preparations produced in the above mentioned way, i.e. not altered by water, which show changes in the above discussed sense, i.e. at one end or on the whole are more or less swollen, irregularly formed, or almost dissolved (puffy).

#### 2. The red sarcina

The previously described culture had been made to a great extent before I had the opportunity of getting to know the literature about the red coloration of the klippfish. Having succeeded in isolating bacteria that showed a red similar to the red coloration of the klippfish and that provoked a corresponding red coloration on the klippfish, I had to believe that I had found the organism responsible for the red coloration. I had, however, occasionally noticed cocci-like bacteria during the microscopic examination, but had considered them to be contaminations. Through the information given by Beckwith and Kellerman about diplococci, or rather micrococci, I was induced to pay closer attention to these cocci.

After an interruption of the studies due to the prevailing circumstances in the fall of 1918, a piece of klippfish lay in front of me, on which a pale pink colored little heap of bacteria was present in great masses. Oddly enough they spread out little although the fish piece had been stored in a closed glass bottle for more than half a year. There was no transfer of red coloration from the bacteria to the fish as well, but the color rather remained reduced to the little pile of bacteria.

On small clay tiles laying on salt with uncooked fish extract it was possible, without difficulties, to bring these bacteria into development, too. With the naked eye, the cultures that came forth were hardly discernible from the red *Bacillus*. They did not appear so clear and translucent, rather they were milky or cheesily turbid, their color though very similar, was a bit paler and fainter, playing somewhat in the pink coloration.

The microscopic examination caused this organism to be recognized as a *Sarcina *(Figure 2, Image 10). One does admittedly on occasion see round individual cells or groups of only two or four cells; usually, however, there are eight or more round cells that are arranged like cube corners to great cube-like or irregular packages lying together, just in the same manner as the familiar *Sarcina ventriculi*. The size of the individual cells varies to quite a large extent, and the sizes of the packages are often noticeably different, too. The findings were 1.5 - 2.2 μ for the cells and up to 15 μ and even more for the packages. I at first did not like to believe that in my culture there still were two different types mixed in; on occasion, however, one could examine the question once more. There is not any coloration recognizable at the level of individual cells or the packages. The color must also be contained in the cells here, since it is completely insoluble and therefore does not diffuse, as will be shown below.

In contrast to the behavior of the red bacilli, it is noteworthy that this *Sarcina *tolerates the transfer from the saturated common salt-containing environment to pure water without any visible damage. This is why one succeeds in producing the preparations using pure water without difficulty. It is possible to get a very strong coloration with Methyl violet 5 B and embedding in Kanadabalsam. One obtains more advantageous images by the embedding in glycerin gelatin which is to be cooled down until it is close to solidifying, because being warm, it draws out the color too much.

The *Sarcina *is negative to Gram-stain. The statement of this behavior at first caused some difficulty, since the *Sarcina *easily retained a bit of the blue-violet color and appeared quite dark after contrast coloration with fuchsine. I did, however, finally obtain convincing colorations if, in a wide spread of Gram-positive staphylococci (the staphylococci were made available to me by the Hygienic Institute, as were the reagents required for the Gram coloration (Anilinwater-Methylviolet, Iodine potassium, Acton alcohol, Fuchsein solution) in an Institute tested composition), I spread *Sarcina*, and at the same time brought in such of the red *Bacillus *and the below described *Micrococcus *and treated all four microbes at the same time. Because of the *Bacillus*, I at first used a fixation with a weak alcoholic iodine solution and not until the washing with alcohol and the drying did the fixation with the flame occur. Then the common salt was washed off with water and afterwards the coloration was carried out in the usual fashion. The preparations made in this fashion showed the *Staphylococcus *dark violet blue, almost black blue colored, the *Bacillus *light red, the *Micrococcus *dark red and the *Sarcina *yet a grade darker, but still clearly red and noticeably different from the staphylococci. Since the staphylococci in this process lie directly next to the other bacteria, the difference is easily and confidently determinable.

The cultures on clay tiles obtained by transfer out of the little pale red colonies available on the fish, consisted without doubt mostly of *Sarcina*. There were smaller quantities of little rods present between the *Sarcina-*packages. It was hardly probable that this mixing was involved in the appearance of the red color; the question, however, needed to be tested by experimentation. Dilution and spreading onto fish agar in Petri dishes resulted primarily in the pale red, turbid-looking *Sarcina-*cultures. Interspersed, there were individual, clear, yellowish drops containing small little rods. One can conclude from this that these small little rods are not the carrier of the red color, but rather it is due to the *Sarcina *itself. The little rods, however, obviously tolerate the saturation of the nutritional bases with common salt, like the *Sarcina *and the red *Bacillus*.

The development of the *Sarcina *is as slow as the one of the red *Bacillus*. Obviously there is inhibition by the common salt in quite the same way. The slow development also corresponds to a lengthy storability. Individual bowls were stored nearly a year long or more.

Attempts were made to culture the *Sarcina *essentially on the same nutritional base which I had used for the red *Bacillus*. I will report in the following about the success subsequent to the list given under *Bacillus*. All the nutrient bases were common salt saturated.

1. Clay tiles on fish pieces or on common salt that was soaked with germ-free fish extract or with fish decoction: development good, color powerful.

2. Common salt soaked with germ-free fish extract or with fish decoction: development slow, but the liquid finally becomes pink and contains *Sarcina*.

3. Fish extract or fish decoction saturated with common salt, but without further additives: white sediment made of *Sarcina-*packages, liquid mainly clear.

4. Agar nutritional base: a) with fish extract or fish decoction: good. Colonies paler red, turbid and not translucent, while those of the *Bacillus *are deeper red, clear and shiny; b) with Liebig's meat extract: not tested; c) with peptone, asparagine and glucose: very weak; d) with Salep decoction: very weak.

5. Albumin: not tested.

6. Gelatin: not tested.

7. Horse serum with 1% peptone and 1% glucose or with peptone alone: also excellently suitable for *Sarcina*. The colonies grow to form thick coatings that only differ from those of the *Bacillus *by the absence of shine and the somewhat paler color.

8. Flour mash (made with corn flour): a) without addition: development weak, but better than that of the *Bacillus*, there develop powerfully red colored colonies; b) with fish extract or fish decoction: development very powerful, strongly red coloration, but the color tone somewhat different from that of the *Bacillus*, going somewhat more towards the pink; c) with Liebig's meat extract: good.

With regard to the way of life of the *Sarcina *and the ways by which the fish can be infected by them in factories, the following observation is of interest. At the end of January 1919 I got news via the fish management office that there was a new appearance of the red coloration, and actually both on some fish from a big shipment of stock fish (unsalted, dried cod) and on the wall of the storage room. The coloration on the fish, however, turned out to be only a contamination with a red color. Herr Prof. Dr. W. Göhlich from the State Chemistry Laboratory, who was so kind as to study the mass chemically, stated that it consisted of an iron oxoid. It would have been apparent if there had been a development of red salt loving bacteria on the unsalted fish. It would have been apparent, if there had been a development on the unsalted fish, of one of the red salt-loving microbes. The red mass on the wall however, really did mainly consist of the red *Sarcina*. Even though this fact had already been determined through microscopic observation and appeared to be highly possible, it turned out to certainty be the result, when a culture was set up on a clay tile. It can be presumed that the walls had already been drenched by the salt and nutrients of the previously stored salt fish up against the walls; they had thus been an appropriate nutrient base. It may be linked with the fact that the *Sarcina *is on the whole less susceptible to the composition of the nutrient base than the *Bacillus*. This will be further illustrated in the corresponding cultures below.

#### 3. The red micrococcus

A third red bacterium was isolated on one of the plaster tiles, which was used to create the first cultures reported above. They contained lively red colonies that grew on spreadings of brownish-yellow bacteria. The culture had been created in November 1916 and in October 1918 and it still clearly showed the red colonies. Since I could not examine all cultures microscopically, and since the colonies appearing in this type and coloration corresponded to those of the red bacilli, I held them to be the latter named one. So it later turned out that the organism in question here is different from the *Bacillus *and the *Sarcina*. It was easy to isolate. It grew well on clay tiles lying on common salt soaked with fish extract and it was extremely beautifully and powerfully colored on horse serum with 1% peptone and 1% glucose or even without the latter. In the same way it was brought to development on corn mash with an addition of fish extract, fish decoction or Liebig's meat extract. The nutritional base compilation described under *Sarcina *is unchanged here. The appearance of the cultures on all these nutritional bases was the same as that of the *Sarcina*, so that only microscopic examination could decide which of the two organisms was present.

The cells are spherically rounded, they have a diameter of 1 to 1.5 μ. If one distributes a sample of a colony in water, the cells partly separate, to a greater degree, however, they stay together in groups of two or four cells. They also like to unite to bigger irregular groups probably during the drying of the preparations. Changes are not observed during the smearing with water, just like in the case with the *Sarcina*. The reaction to Gram-coloration is negative. The proof was obtained in such a way that the micrococci were smeared and colored with Gram-positive staphylococci next to and upon one another on the same object slide, whereby both bacteria could be directly compared. The staphylococci were dark blue; the micrococci of the klippfish were strongly red colored. Compare the data under *Sarcina*.

#### 4. Further strongly colored bacteria

In the cultures I prepared in the summer of 1918 out of the available red cultures by smearing on horse serum (see above), there appeared a colony with a noticeably carmine-red color. It quickly reached an expansion of about 2 cm in diameter, apparently at first assisted by the spreading of a threadlike fungus, which took the bacteria along and later on grew beyond their range. It was noticeable that the surface of the culture was not oily and shiny, but matt and appeared almost dry. The microscopic study resulted in roundish-oval single cocci of about 0.6 - 1.2 : 0.5 - 0.8 μ in size.

One tried to continue cultivating this organism, on the different types of nutritional bases that were used during the studies, but unfortunately without success. Also the transfer onto horse serum which was similar - as far as I could evaluate - to the original nutritional bases, was unsuccessful.

Furthermore, another organism of a deep orange-yellow color was found. Since here too, the further culturing caused difficulties and the color tone was essentially different, I distanced myself from further, more exact research. They were small roundish cocci of 0.5 - 0.6 μ in diameter.

### C. Comparison of the found bacteria with the previously observed ones

The specification of the previous observers about the nature and cause of the red coloration of the klippfish are confused and contradictory. It must be examined if it is possible to make them consistent with one another and with the facts of my findings.

I did not find any organism corresponding to the *Clathrocystis roseo-persicina*, which according to Farlow is the cause of the red coloration and which also according to Patouillard, Heckel and Beckwith is supposed to occur on the klippfish. According to the information from Zopf, the *Clathrocystis *can create pink red, intensively blue red or violet coatings on rotting animal and plant material and it can live in fresh and salt water. Based on that, it would therefore not be completely impossible that it occasionally would make coatings on klippfish. But it cannot be considered as a cause of the actual and commonly appearing red coloration of the klippfish.

I think it is quite probable that the *Sarcina *observed by me corresponds to the *Sarcina morrhuae *of Farlow. According to Farlow, this *Sarcina *is, however, supposed to be colorless, but Farlow did not cultivate it. The individual cells and cell packages of my *Sarcina *appear colorless, too, as mentioned above; greater quantities together, however, develop a vivid red, often even an intensively red color, and on several specimens of the klippfish at hand (compare below), I have determined that this *Sarcina *is definitely the carrier of the existing red. The little colonies sat as red small dots or droplets on the fish flesh, which in general was brownish yellow colored.

It is very doubtful to me that Farlow's *Sarcina morrhuae *and Poulsen's *Sarcina litoralis *are identical, as indicated by Farlow, since firstly, the drawings of Poulsen in no way resemble anything like a *Sarcina*, and secondly the nutrient base on which the *Sarcina litoralis *was found is an essentially different one.

Mégnin's *Coniothecium Bertherandi *and Layet's sarkoden-like organisms could perhaps correspond to the *Sarcina *although the descriptions and especially the completely false indications of the size of the *Coniothecium*, only allow one to guess. Johan-Olsen's *Sarcina rosaceae *has substantially smaller cells.

It is difficult to get a clear picture out of Høye's repeated publications. In his publication of 1904 he mentions the red *Sarcina*, which he believes is one of the causes of the spoilage of the klippfish. At the same time and later on, he describes forms belonging to the still lesser known genus *Sarcinomyces *that in certain stages allow a confusion with *Sarcina*.

The micrococci and diplococci described by Høye, Beckwith and Kellerman seem to be more or less *Sarcina-*like organisms. Kellerman believes Beckwith's *Diplococcus gadidarum *and Høye's *Micrococcus *even to be identical to *Sarcina litoralis*. But this cannot be right simply because of the fact that Beckwith observed the *Diplococcus *in addition to the *Sarcina*. Besides, the cells of those *Diplococci *and *Micrococci *are substantially smaller than those of the *Sarcina; *they are supposed to be present individually or joined in pairs, not to occur as packages and according to Beckwith and Kellerman, they act positive with Gram coloration, whereas the *Sarcina *is Gram-negative according to my observations. That is why Kellerman's *Micrococcus litoralis*, which is considerably smaller than the *Sarcina*, cannot correspond to the latter. Therefore, the cocci described by Beckwith and Kellerman do not have anything to do with the *Sarcina*.

In contrast the similarity with which Beckwith's *Diplococcus gadidarum *and Kellerman's *Micrococcus gadidarum *show with the red *Micrococcus *isolated by me, is noticeable, specifically regarding the oddity that the cells often remain stuck to one another in pairs, not seldom in foursomes. According to the cell size (1 to 1.5 μ) my *Micrococcus *corresponds to the *M. litoralis *of Kellerman, while *M. litoralis gadidarum *that is supposed to correspond to Beckwith's *Diplococcus gadidarum*, is not half as big. But nevertheless my *Micrococcus *must be different from Kellerman's, since this one is supposed to be Gram-positive, while my *Micrococcus *is negative. Specifically because of this fact I concerned myself with carefully ascertaining its reaction to the Gram coloration and its immediate comparison with Gram-positive bacteria.

With regard to the bacilli, too, one cannot reach a certain conclusion. Edington's *Bacillus rubescens *squares with the size of the *Bacillus *I found. The information is notable, too, that it grows well on flour mush (apparently, however, without the addition of fish extract) and that it becomes strongly red, while other nutrient bases are less suitable. But this information alone cannot lead to an accordance, all the less so, since Edington gave no further information about the constitution of this nutrient base of flour mush and especially not whether the nutrient base was salty and to what degree or not. The information of Edington that both the little rods and the longer threads form spores speaks against the accordance. Also the description of the cultures growing on agar does not sit well with the appearance of the *Bacillus *I found, when it grows on agar. Regarding this, Edington says: "On Agar-Agar it does not succeed well at the ordinary temperature, but if incubated it forms large films over the whole surface of a grayish color, and which begets the appearance seen in the oil-painted surface which has been exposed to the sun, i.e. cracked and blistered." Cultures of my *Bacillus *on agar when flowing together looked like a freshly lacquered surface, not cracked or ripped and not grey, but always clearly red, often even strongly red. Edington does not mention any other characteristics that would be distinguishing. Therefore there is no sufficient ground of surmising that the *Bacillus *I found corresponded to the *Bacillus rubescens *of Edington.

Le Dantec has described two supposedly different bacilli one after the other and at first declared the one and afterwards the other one as the cause of the red coloration. According to their length, they both could correspond to the *Bacillus *isolated by me; the thickness is not given. Both are hard to bring to development and they depend on specific conditions of their nutritional base. The first, the "red *Bacillus *of Newfoundland", *Bacillus Danteci *Krause, appeared in a manner similar to the way it did in my cultures (compare Section IV), i.e. in the form of red drops on colonies of whitish bacteria. It developed poorly and without noticeably red coloration in liquid nutrient base. At one end of the little rods there was supposed to be almost always present a shining spore. This circumstance vividly reminded me of my observations made of the bacilli in salt solution. It was already exposed above that the shining points in my culture were not spores. I did not at all find any real spores. Le Dantec, however, further reported that he used the resistance of the spores to high degrees of heat in order to create pure cultures. That is why one could conclude that there were indeed spores. So "the red Newfoundlander" would have to be different from the *Bacillus *I have at present. The second *Bacillus of *Le Dantec, the "microbe of the klippfish's red coloration" seems - as far as comparison characteristics are present - much more to agree with the one I isolated. Like this one, it comes to development without trouble on common salt oversaturated nutrient bases. Like this one, it is Gram-negative and forms no spores. There is, however, a different piece of information about the development of a red veil after 5 to 10 days on liquid nutritional base (bouillon), a manifestation I could not observe. Le Dantec did not make any observations about its behavior towards water. Although it cannot be said with certainty, I really think it to be possible that the *Bacillus *isolated by me corresponds to the second microbe of Le Dantec, to the "microbe du rouge de morue". If Le Dantec's own comments did not contradict it, one could even presume that the first *Bacillus *also was the same one.

The cocci-like bacilli of Peirce's and Le Dantec's "Coccus du rouge de morue" still remain quite puzzling. According to his descriptions, the illustrations of his cultures and their appearance on salt, one could surmise that Peirce had in front of him one of the above mentioned red colored bacterial types. But one cannot find out more, since the dimensions given neither fit the *Bacillus*, nor the *Sarcina*. More precise descriptions and illustrations of cells, from which one could deduce, are not given and the information that the red color material diffuses out of the colonies contradicts the experiences I had with the bacteria I observed. Le Dantec's *Coccus*, too, has sizes that are considerably beyond those of the *Sarcina*.

I summarize the results of the previously noted considerations as follows:

1. The *Sarcina *I found corresponds to the bacteria mentioned and described as *Sarcina *by earlier observers, especially by Farlow. It is a true *Sarcina *and has nothing to do with Beckwith's and Kellerman's described diplococci and micrococci. I choose for it the name given by Farlow, ***Sarcina morrhuae***, since I could not convince myself that Poulsen's *Sarcina litoralis *is the same organism.

2. According to size and appearance the red *Micrococcus *is in accordance with the cocci labeled by Kellerman as *Micrococcus litoralis*. Since, however, it is Gram-negative, it must be different from *M. litoralis - *unless Kellerman's information is deficient - and therefore it must also be named differently, all the more so as the name given by Kellerman was given on the basis of a wrong assumption. I call it ***Micrococcus*(*Diplococcus*) *morrhuae***.

3. The red *Bacillus *with great probability corresponds with the second of Le Dantec's described bacilli. It cannot be determined if it has a relationship with the first *Bacillus *of Le Dantec (*Bacillus Danteci Krause*) as well as with Edington's *Bacillus rubescens*. Since Le Dantec only named his bacilli in French, I call it ***Bacillus halobius ruber ***recognizing its prominent characteristics.

4. Because of the multiple lack of correlation between the information of the earlier observers and my own observations the question remains to be debated in the future, if, besides the three above described, there are further bacteria which have a preference for strongly salty nutritional bases in connection with the creation of lively red coloration and if they occasionally occur on the klippfish at the same time. The information given above of the earlier observers would in this way have to be examined. One can specifically point to Beckwith's *Diplococcus gadidarum *and the bacilli of Peirce, which according to their size do not correspond to any of the above described types. I myself presume that the carmine red oval cocci, which I unfortunately could not examine further, are a further kind of this type of organism.

### D. The adaptation to high salt content

Especially noteworthy is the appearance that these bacteria live on nutritional bases with high salt concentration. It was already mentioned that the colonies occasionally sit directly on the salt crystals excreted by the nutritional base. As is known, common salt is frequently used as a material to inhibit the development of microorganisms and therefore it is antiseptical and conserving. The effect might be, at least to a part, due to the high osmotic pressure of stronger or more saturated solutions. In contrast, it is to be stated that certain organisms tolerate higher salt concentrations or even prefer them.

One will have to distinguish between the ability to resist salt solutions, the adaptability to stronger concentrations and the preference for strongly salt containing nutritional bases or the exclusive adaptation to them.

The ability to resist salt solutions has particularly been researched with regard to the practically important question how long certain bacteria known as damaging ones remain viable when the flesh is salted. There are reports at hand by Koch [77], de Freitag [78], Stadler [79], Peterson [80], Lewandowsky [81], Müller [82], Weichel [83], Serkowski and Tomczak [84], v. Karaffa-Korbutt [85], Reimers [86] and so on.

In general there is a strong inhibitory effect on the development on salt-containing nutritional base, already at weaker salt content, too. Killing off of the bacteria often occurs, however, even in concentrated solutions, and often not until after several weeks or months. The spores withstand even longer. According to the researches by Müller, they were still viable after two years.

One can speak of the adaptability of such organisms that, though they indeed usually live on salt-poor bases, are also capable to prosper even on salt containing ones. This is most easily demonstrated by the mold of the family *Penicillium*. When during my experimentation contamination with them occurred, they came into considerable development despite the saturation of the nutritional base with common salt. The growth was particularly strong on the nutritional bases of horse serum, and it may still be that their other factor, (perhaps their content of powerfully acting nutrients) is of supporting importance. Also a number of bacteria, that were not further studied, reached a more or less rich development on the strongly salt containing nutrient base.

There are already studies available about several questions following the adaptation.

According to A. Fischer ([87] but I have only seen 1. Aufl.), the permeability of the bacteria to salt solutions is supposed to be a determining factor, as to whether they quickly and without damage, adapt to higher concentrations. The permeable bacteria are accordingly less sensitive to higher concentrations.

Organisms that cannot easily adapt to higher salt concentrations can occasionally become used to it in the course of several generations. There ought to be a selection of the most adaptable individuals which under the different conditions, continue to propagate alone, whereby, in the course of generations, the development of one adapted race comes into being, in which the new quality is somewhat hereditary.

Eschenhagen [88] indicates that the fungal conoids that arose on concentrated nutrient base only germinate on concentrated ones.

Errera [89] tries to refer the adaptation, that *Aspergillus*-conoids show to the medium that had carried the fungus, back to the inheritance of the ability of producing osmotically potent substances (la faculté de produire, en cas de besoin, une plus forte turgescence).

Laurent [90] and Clerfeyt [91,92] found that yeast through longer culturing, can be made used to higher salt contents of the nutrient base. It was possible to obtain adaptation to pressures up to 60 and 80 atmospheres. Along with the adaptation to high pressures, which go hand in hand with the slowing of growth, rounding of the form, reduction in size, disappearance of the vacuoles, greater tendency to build packages, richer production of glycogen, slowing of fermentation. These changes can be reversed by culturing on ordinary nutrient base. According to both authors the osmotic pressure is not solely critical for the development, but in addition, the type of salt, especially of the base has impact. Yeast that has adapted to potassium nitrate or potassium sulfate is supposed to have grown the best in the presence of potassium nitrate and respectively of potassium sulfate; and it is supposed to have even grown better on potassium salts than on sodium- calcium- or magnesium salts.

With regard to the previously mentioned cases concerning the red bacteria of the klippfish, namely concerning the *Bacillus halobius ruber*, there exists not only apreference of strongly salt containing nutrient bases, but partly even an exclusive adaptation to such an extent that the development only occurs at high salt concentrations. The experiments described in the preceding have already shown that it is possible to obtain quite pure cultures of the red bacteria on common salt saturated nutrient bases (if they are otherwise of suitable composition) without difficulty, whereas salt free or salt poor nutrient bases fail. It was possible to be even more sure of this result through the use of nutrient base with tiered common salt content. For this purpose cultures were prepared on the agar slants in test tubes. It was already emphasized in the preceding that despite the existing adaptation, the development goes forth extraordinarily slow.

Experiment 1. 27 glasses each with about 7 ccm corn-mash-agar and an addition of common salt of 0, 1, 2, and so on up to 26 dg. Salt content accordingly of 0 to 37%. At first the glasses 26 to 20 were inoculated with a culture of the red *Bacillus *from a culture on clay tiles, later the glasses 19 to 15 out of 20, the following out of 15 and so forth. The development was the strongest at the highest concentrations (26 to 20) and strongly stepped down as the concentration dropped. From glass 8 downwards there was no more detectable growth. The nutrient base is not particularly suitable.

Experiment 2. 14 glasses each with 5 ccm agar with decoction from fresh fish and salt addition of 0, 1, 2, and so on up to 13 dg (0 to 26%). Red *Bacillus*. Result: weak development starting at 6 dg, from 9 dg onward more powerful, 11 to 13 strongly red.

Experiment 3. 8 glasses each with 5 ccm nutrient agar from the Hygienic Institute (yeast extract as replacement for bouillon) with salt addition of 1, 3, 5, and so on to 15 dg (2 to 30%). Red *Bacillus*. Development only at 15 dg somewhat powerful, at 13 weak, till 9 downwards unclear traces. Nutrient base apparently not very suitable.

Experiment 4. 7 glasses with the same nutrient base and 2, 4, and so on to 14 dg of salt addition (4 to 28%). *Sarcina*. Development at 2 and 4 very weak, 6 to 12 powerful, 14 less strong.

The experiments show that these bacteria absolutely prefer a high salt content, but even come to development on medium ones, but that in contrast nutrient bases without common salt or such with lower common salt content are unsuitable for the development. The lower limit for the *Bacillus *lies, as far as the few experiments allow us to conclude, at a salt content of 12%. It seems impossible to get it used to stepwise lower salt contents. The *Sarcina *grows even on a nutrient base with lower content. Apparently the remaining composition of the nutrient base also has an influence on the minimum at which growth still occurs.

The adaptation to high salt content requires a simultaneous adaptation to osmotic pressure. The bacterium cell must either be capable of letting through whatever salt quantities, so that the same salt concentration is created in the cell as is present outside, or the cell must produce other osmotically effective substances that at once bring forth pressure which is in equilibrium with the outer pressure. The first of these possibilities is presumably the more probable one. It is hard to say whether the matter is explained therewith. It is possible to answer the question more certainly, if the common salt regarding its osmotic pressure or its otherwise effect on the bacterial cells can be more or less replaced by other substances. Experiments similar to the ones just mentioned were set up, in which the lowering down of the highest concentration of the common salt content corresponded to the rising addition of an osmotically potent substance. Since at first they were supposed to be preliminary experiments, one did not care about calculating and exactly weighing the salt amounts that would have been necessary to create the same osmotic pressure for all experiments. Unfortunately, exact experimentations remained undone, as a result of the occurring lack of appropriate fresh fish. Klippfish is not usable due to its high salt content, and the decoctions of the fresh fish that were available at the time, did not seem to suit the *Bacillus*; so a series of experiments remained completely without any result. Hitherto, other work has prevented the restarting of the experiments. In order to be able to consider the error founded on the process I have calculated the quantities of the common salt which are equimolecular to the added amounts of the other salts; and in each experiment I have indicated the common salt amount equimolecular to the whole salt quantity. Not taken into account is the potentially different ionization of the salts that, in the present circumstances, is hard to judge.

Experiment 5. Sodium nitrate. Red *Bacillus*. 12 glasses each with 5 ccm agar with fish decoction and altogether 30% addition of salt. The salt quantities are individually given in decigrams in the two following lines. The third line gives the amount of the common salt equimolecular to the total salt quantity. The same is true for the successive experiments.

Result: No development in NaCl 2 to 7, traces in 6 and 8, powerful development in 9 to 13.

Experiment 6. Sodium nitrate. Red *Bacillus*. 14 glasses, the same nutrient base. Salt addition 26%.

Result: No development in NaCl 0 to 5, weak in 6, rising in the following, especially strong in 11 and 12.

Experiment 7. Potassium chloride. Red *Bacillus*. 12 glasses, the same nutritional base. Salt addition 30%.

Result: No development in NaCl 2, only traces in 3 and 4, little in 5, plenty in 6 to 13.

Experiment 8. Sodium nitrate. *Sarcina*. 9 glasses, agar with meat decoction. Salt addition 32%.

Result for an unsolved reason not clear: No development in NaCl 0 to 4 and 10 to 14, abundant in 6, 8 and 16.

Experiment 9. Potassium nitrate. *Sarcina*. 9 glasses, the same agar, salt addition 32%.

Result: No development in NaCl 0 to 12, abundant in 14 and 16.

Experiment 10. Potassium chloride. *Sarcina*. 9 glasses, the same agar, salt addition 32%.

Result: No development in NaCl 0 to 4, clear development in 6 and 8, abundant in 10 and 12, somewhat weaker in 14 and 16.

Experiments corresponding to experiments 8-10 with the red *Bacillus *remained completely without any result. Apparently the fish decoction used for these 6 experiments was unsuitable for the *Bacillus *which is much more sensitive than the *Sarcina*.

If only the osmotic pressure of the nutrient base were determining for the development of the bacteria, in these experiments approximately the same development should have occurred in all the glasses. Since this was not the case, a specific effect of the individual salt types must be present, that is to say, the common salt or the ions into which it is disassociated, cannot be replaced by other salts or their components. The three salts tested NaNO_3_, KNO_3_, and KCl in themselves do not seem poisonous or particularly inhibitory in their effect on bacteria, at least not in the middle and weaker concentrations, since the *Bacillus *comes to development even upon addition of 7 dg NaNO_3 _(14%) or 10 dg KCl (20%), and the *Sarcina *even upon addition of 2 dg KNO_3 _(4%) or 10 dg KCl (20%). In contrast, however, it seems that the stronger concentrations in some cases inhibit strongly.

This result is shown more clearly in a series of further experiments, in which the NaCl-concentration for all glasses was kept the same and only the quantities of the other salts was tiered.

Experiment 11. Sodium nitrate. *Sarcina*. 9 glasses each with 5 ccm agar with decoction of fresh fish and each with 12 dg NaCl. In addition:

Result: abundant development with NaNO_3 _0 and 2, weaker at 4 to 8, weak at 10 and 12, none at 14 and 16.

Experiment 12. Potassium nitrate. *Sarcina*. 9 glasses, the same nutritional base, additions added to each besides 12 dg NaCl:

Result: Powerful development at KNO_3_, 0 to 4, good, but less abundant at 6 - 14, weak at 16.

Experiment 13. Potassium chloride. *Sarcina*. 9 glasses, the same nutritional base, additions added to each besides 12 dg NaCl:

Result: Powerful development at KCl 2 to 6, no development at 8 to 14 and in a conspicuous way at 0.

Three series of experiments with the red bacillus in tubes that were made with the same nutrient base and the same additions remained without result, since the *Bacillus *did not grow.

Accordingly, the *Sarcina *tolerates about 24% sodium nitrate, 28% potassium nitrate, 12% potassium chloride.

As already indicated, these experiments would require repetition and completion, as they were only supposed to be preliminary experiments and to an extent did not succeed. Especially the numbers cannot claim to be precise. But in the meantime the following conclusions can be derived:

The flourishing of the salt loving bacteria is not dependent upon the osmotic pressure of the nutrient base alone, but rather also on a specific impact of the salts or their ions. Sodium cannot be substituted by potassium. The requirements of the *Bacillus *and those of the *Sarcina *regarding the salt content are different. The *Bacillus *is adapted to high and highest contents, it seems as if it cannot be adapted to lower ones. The *Sarcina *tolerates the highest contents, however, flourishes at lower ones, too. Potassium and the nitrate group, regarding the ions, do on the one hand not bring forth a poisonous effect, but on the other hand inhibit the growth at middle and particularly at high contents.

In a similar vein, Laewandowski [93] reports about experiments, though he has admittedly not worked with highly salt loving, but rather with salt tolerant bacteria. He considers the growth inhibition mainly to be as a consequence of the high osmotic pressure. It is at the same time influenced by a specific ionic effect, since it turns out that sodium salts inhibit more strongly than the corresponding potassium salts. On nutrient bases with more than 25% common salt there was no more growth. In this respect the behavior of these bacteria substantially differs from the salt loving ones. A preceding culture in bouillon containing high salt concentrations did not make the bacteria more suitable for growth on highly concentrated nutritional base than they were before.

### **E. Sensitivity to changes of concentration "plasmoptysis**"

The adaptability to high salt contents is in remarkable contrast with the sensitivity to changes of concentration that appears to such a conspicuous degree with the red *Bacillus*.

If bacteria grown on common salt saturated nutritional base are suddenly transferred into pure water, this is indeed such a precipitous change of the external conditions and it must be connected to such a forceful disturbing of the balance between the osmotic inner pressure and the pressure of the surrounding liquid, that the momentary bursting and dissolving of the cells cannot be surprising. One must be even astounded that the sarcinae and micrococci appear to stand this alternation without visible change. It remains to be seen, if the assumption of a high degree of permeability of the *Sarcina *for the common salt explains the difference.

However, there are observations existent, that a far lesser change of pressure acts detrimentally on bacterial cells. According to A. Fischer [94] the bactericidal characteristics of certain sera do not rely on specific poisons, but rather on changed osmotic pressure. Fischer found that a change in salt content of the nutrient base of no more than 2% brought forth alternations that are quite similar to the observations made by me of the above described dissolving appearances of the red bacilli or perhaps correspond to them entirely. He assumes that they are the result of a raised internal pressure. A part of the protoplasma or even the whole protoplasma is supposed to be pressed out at places where the flagella arise, or to be pressed out by rips occurring in the membrane. To be precise, at first they are pressed out in form of droplets or small balls, which soon thereafter die off and dissolve. He calls the phenomenon "Plasmoptyse". Particularly little rod formations are supposed to be subject to the changes, while the cocci are more resistant. This also corresponds with my own findings.

It is completely believable that the appearances observed by Fischer can occur through transfer of the bacteria into solutions of lower salt content. This explanation arose easily for the above described changes of the bacilli of the red klippfish. It also makes comprehensible the great resistance of coccal forms apparently because the surface area compared to that of the cylindrical little rods, which is much greater in comparison to their volume, makes possible a much faster assertion of the pressure difference than the spherical surface. It cannot be surprising that the changes in Fischer's experiments took more time, 15 to 100 minutes, while they in my experiment occurred immediately, if one takes into account the great difference of the osmotic pressure in question.

The statement by Fischer remains, however, conspicuous that the transfer of the bacteria in solutions of higher salt content (from 0.75% to 2%) is supposed to cause "plasmoptysis". I am not able to judge about the observations themselves. But the explanation Fischer gives is not comprehensible. He assumes and even tries to reckon that as a result of the greater surface of the little rods there a surplus of common salt is taken up. As a consequence an internal pressure that goes beyond the pressure of the external fluids is supposed to arise. This is not comprehensible because according to physical principles the penetration of common salt must stop as soon as the internal pressure reaches the same value as the outer pressure or as soon as the concentration of the salt solution is the same on both sides of the protoplasma membrane. If the observation is correct, another point must be looked for [95]. Fischer's explanation also appears to me to contradict his own opinion expressed at another place that the resistant bacteria - and especially the spherical forms that belong to them - let the salt solution pass through particularly easily.

Already, Arthur Meyer [96] has confronted Fischer. He even goes so far as to declare the plasmoptysis as a "child of Fischer's fantasy". He himself observed the swelling of the little rods of *Bacillus cylindricus*, which turned through an intermediate pear shaped condition, into spheres. The experimental description does not allow one to recognize if osmotic powers were effective; in contrast Meyer points toward infringements found in Fischer's work against the theory of the osmosis. In his response Fischer [97] claims that the rounded spheres observed by Meyer are created by the souring of the nutritional base and can be converted back by ammonia fumes. He holds his position that "Plasmoptyse" is a process of its own kind that is connected with the bursting of the membrane. Garbowski [98] too, though as it appears, in a work done under Fischer's influence, distinguishes the rounding and the plasmoptysis as two separate appearances. The pin-like and the two-legged or Cyclops-like, that is to say bent little rods, are supposed to be somewhat different than the spheres without appendices; the latter of which, do not come into being by stripping off of the adhering little rod membrane. The short essay by Leuchs [99] does not bring forth essentially new viewpoints.

I cannot agree with Fischer's opinion that the protoplasma is pressed out through the pores of the flagella or by rips in the membrane. Firstly the *Bacillus halobius ruber *has no flagella, and secondly one does not see smaller or larger droplets or spheres outside the cells as their appendices. The appearance by all means makes the impression of a swelling, that now beginning at a specific place, now on the spot, gripping the whole little rod, and finally causes a dissolution of the whole cell. I imagine that the wall of the little rod is more or less flexible, that the protoplasma fills the cell completely and that the cell juice, which is to be regarded as the carrier of the osmotic pressure, is distributed in the protoplasma in the form of tiniest droplets or perhaps just imbibed in the protoplasma. Under these circumstances the change itself seems to me to be easily understandable. Where the spheres appeared in the preparations, they were little rods which were completely swollen. I could not find membrane fragments in the vicinity of the altered bacilli. There was also nothing to be seen in the threadlike jelly that is the final result of the treatment with water. When wet, it only allows the detection of tiniest microsomal, crumbly components. A little dried and colored, it does not show anything but the above described and, in Image 9, Figure 2 pictured thread or net forming coagulum with microsomal little grains in the threads. However, I must note that the bacilli are very small and that the membranes would be at the limit of performance ability of the microscope.

Upon looking through the preparations of the red *Bacillus*, I note that in almost all of them there are individual cells present that are shorter and thicker or are swollen at one end in a club-like form or somewhere are equipped with a swelling and, at this place, often also bent. They thus show appearances that completely correspond to the above described changes (see Images 3 and 4, Figure 1). The question arises, if these changes already occur occasionally in the culture itself or if they only occur during the preparation. As noted above, the preparation does not work well without a mixture of the colonies with salt solution and as a consequence is not possible without disturbance. The latter presumption is perhaps for the time being the more probable.

In cultures becoming older, namely in those on serum, the transition of the little rods into coccal-like conditions is, however, to be regarded as a degeneration, as an appearance of age or as a result of the culturing conditions getting gradually unfavorable. These structures probably correspond to what in other cases, has been described as involution forms. They remain, at least partially, viable. New sowing onto clay tiles gave a consistent further development and established the normal growth-form again.

I summarize my verdict about the available appearances as follows:

Changes in the manner of plasmoptysis of Fischer to *Bacillus halobius ruber*, are the result of the high osmotic pressure in the cells that makes itself felt as overpressure, when they are brought into water or into salt solutions with lower content. Their nature is not a squirting out of the protoplasma, but a partial or complete swelling or dissolving of the cells. The pressure difference is so great for *Bacillus halobius ruber *that the change occurs immediately, while the plasmoptyse appearances of Fischer take up a longer time, 15 to 100 minutes.

Some works that apply to related subjects might briefly be further mentioned. Braem [100] examined the effect of distilled water on pathogenic bacteria. Swelling, appearance of bottle- and club-forms, vacuolar degeneration, indentation on the border, loss of coloration and similar abnormal appearances were observed that partially, more or less corresponded to the above mentioned ones. According to Matzuschita [101] on the contrary, the common salt content of the nutrient base, namely at contents of 3 to 8%, brings forth certain changes of the figures of the bacteria both on little rods and on cocci. Finally, Lewandowski [81] reports that the bacilli do not change their form in strongly salt containing bouillon.

One can only refer to the part of the subsequent literature that discusses the subject concerning the cause of the bactericidal properties of certain sera [102-105] which has already been broached in Fischer's first publications. The observers tend, in general, to assume specific poisons to be the cause rather than the osmotic disturbances according more in the manner of Fischer.

### F. The red pigments

A second common and very apparent oddity of the bacteria at hand is the creation of the red pigment.

The pigments of the three bacteria are, in several aspects, similar to one another. The difference in tone is so insignificant that it is therefore not possible to distinguish between the three kinds. The different appearance of the colonies of the *Bacillus *on the one hand, of the *Sarcina *and the *Micrococcus on *the other hand is mainly due to the degree of the transparence of the bacterial mass and not or barely to the color tone. There is agreement, too, as far as that the pigment is bound to the cells, and it is not expelled out of them and does not diffuse into the surrounding nutrient base. Upon microscopic examination, the individual cells appear colorless; only larger quantities, densely pushed together show a noticeable coloration. Particularly notable is the behavior of the same kind towards concentrated sulfuric acid. If one takes a small amount of a fresh culture into a drop of this reagent, it surrounds itself with a deep sky-blue foam where the acid has an effect on it. After a few minutes, however, it disappears again and gives way to a dirty brown-violet coloration. Precipitation of blue crystals was not noted during this process. The unavoidable common salt content of the bacterial mass disturbs in this experiment, causing the excretion of bubbles of hydrogen chloride. The behavior is of the same kind towards several other reagents too. At ordinary temperatures caustic potash affected a noticeable paling only after 24 to 28 hours. Hydrogen peroxide or saltpeter acid destroy the pigments quickly. Sulfur dioxide in watery solution discolors at first to pink and destroys the color only after some days.

The only, however, very essential difference I could realize so far consists of their conditions of solubility. The pigment of the *Bacillus *can be easily extracted by cooking with ethyl alcohol or with methyl alcohol. There results a prettily red orange colored solution even with a relatively little amount. Upon heating with water or with propyl alcohol only a little bit of pigment dissolves. After the evaporation of the alcoholic or methyl alcoholic solution there remains the pigment in strongly red droplets in addition to the common salt dissolved as well, that deposits as crystals. The pigment does not seem to crystallize. The droplets easily dissolve in ether, acetone, chloroform, tetrachlorocarbon, benzol, toluol, xylol, petrol ether, benzene, ligroin, sulfur carbonate, clove oil, also in polyalcohol, but only little in water. Upon addition of strong (not completely concentrated) sulfuric acid they color blue from the border inwards, like the bacterial colonies themselves. However, they break down easily, and one must, if possible, prepare it fresh. I therefore could only test a limited number of solvents. The pigment of the *Sarcina *seems to be in contrast completely insoluble in common solvents. I cooked the bacterial mass in water, methyl alcohol, ethyl alcohol, propyl alcohol, vinegar ether, petroleum, sulfur carbon, xylol, pyridine, benzol, and then let them sit in the liquid for two days without a noticeable coloration of the solvents or a bleaching of the bacteria. I furthermore smeared smaller amounts on cover slips, and for several days laid them in tetrachlorcarbonate, trichloroethylene, perchlorethylene, acetone, amyl acetate, aniline, nitrobenzol, clove oil without a noticeable bleaching. Tetrachlorethane, chloroform, vinegar ether did not color as well, although the color of the bacteria seemed to bleach a little. In acetic acid, phenol and cresol there was a bleaching, but these substances might rather decompose than dissolve. I continued to try to bring the pigment into solution by cooking in a mix of methyl alcohol, ethyl alcohol and hydrochloric acid or methyl alcohol, ethyl alcohol and potassium or after precursory treatment of the bacterial mass with potassium or hydrochloric acid in alcohol, but equally without success.

Most of these experiments were also performed with cultures of the *Micrococcus *and had the same negative result. The pigment of the *Micrococcus *is obviously the same or similar to that of the *Sarcina*.

In order to be able to examine the pigments of the *Bacillus *spectroscopically, I have collected the bacterial mass out of a great number of cultures and extracted it in a Soxhlet-Apparatus with alcohol. The examination of the solution at the Physical State Laboratory done with friendly help on the part of Herr Prof. Dr. B. Walter by the use of a big spectral apparatus gave 3 absorption bands in the spectrum, namely

1. in the green,   Maximum at 528 μμ, powerful,

2. in the blue green,   Maximum at 493 μμ, very powerful,

3. in the blue,   Maximum at 462 μμ, weak.

The middle of the first band approximately coincides with the Fraunhofer Line E (527 μμ), that of the second lies in front of F (485 μμ), that of the third between F and G (429 μμ) closer, however, to F.

Since the conditions of solubility of the red pigment of the *Bacillus *and the behavior to sulfuric acid seem to point toward relations to the lipochromes (see below), I tried to obtain the pigment in a purer form by saponifying a part of the alcoholic solution with some potassium and by shaking with solvents the mass taken up with water. But I did not have any success. The ether remained colorless, the toluol only colored weakly yellowish. When the rest of the alcoholic solution mixed with a bit of potassium was evaporated, there was a separation of similar red droplets in the brownish residue, as was the case at the immediate evaporation of the solution. The potassium apparently only had a little effect. Further experiments remained for the time being undone due to lack of sufficient material.

Since the pigments of the *Sarcina *and the *Micrococcus *are insoluble, I made the attempt of studying their color in striking light. I irradiated the colonies with direct sunlight and used a Seibert Microspectral apparatus. A dark absorption band was found in the green, about between 555 and 535 μμ, and general darkening at about 515 μμ and downward. Due to insufficient brightness, it could not be ascertained, if, in the darkened part at about 502 μμ, there is a further dark band. It was just as little possibility to find out if there were differences between the three bacteria in the position of the darker parts. The experiment confirms for the time being, only the great similarity of the pigment tone.

The development of pigments by bacteria is a known and widespread phenomenon. It appears desirable to compare the discovered facts in the preceding - although being rare - with what is existing in the literature about similar pigments. One tried to make groups of the pigments according to their behavior. Beijernick [106] distinguishes three types of bacteria: firstly chromophore bacteria in which the pigment makes up an integral component of the cells similar to the chlorophyll of the higher plants, secondly the chromophore bacteria, the real pigment bacteria, in which the pigment is exuded as useless excrete and that under certain circumstances can live without development of color and thirdly the parachromophore bacteria, for which the pigment is also an excretory product, but it sticks to the cells. According to this division one would probably have to place all three of the present bacteria into the first group. There is, in the meantime, no reason to believe that the pigments play an important role in the cell life, somewhat like the chlorophyll or perhaps the bacteriopurpurin. Migula [107] divides the bacteria into three types: firstly the bacteria, the pigments of which dissolve in water, secondly the bacteria, which have pigments that do not dissolve in water, but in alcohol or in fat solvents such as ether, benzol, sulfur carbon etc. and thirdly the bacteria the pigments of which dissolve neither in water nor in alcohol or in fat solvents. The red *Bacillus *must be put into the second group, its pigment is hard to dissolve in water, but easy to dissolve in fat solvents. The third group, of which only a few examples (Migula only names *Micrococcus cereus flavus*, pigmentation yellow, extractable by a 10% cooking potash, and *Pseudomonas berolinensis*, pigment soluble in hydrochloric acid, the solution is not sustainable. See also, Neumeister, [108]) are known, gets a notable enrichment by the *Sarcina *and the *Micrococcus*. But at the same time the whole division seems to be schematic and unnatural.

The pigments of the second group of Migula belong partly to the lipochromes or fat pigments that are also spread out elsewhere in the plant and animal kingdoms. The blue coloration with concentrated sulfuric acid is a common feature of the pigments next to the conditions of solubility, that in detail are different, and next to the circumstance that in general the pigments are bound to fats, of which they can be separated by saponifying.

The carotene, C_40_H_56_, forms crystals, is insoluble in water, hard to dissolve in alcohol and acetone, easier in petroleum ether, very easy in sulfur carbon. The crystals are colored indigo blue with concentrated hydrochloric acid. The spectrum shows absorbance bands in the blue green and blue, according to Willstätter and Mieg [109] at 488 to 470 and 456 to 438 μμ, according to Tswett [110] at 492 to 475 and 460 to 445 μμ. Other observers give still other descriptions; perhaps they studied other or not completely pure substances. Tswett [111] presumes that there are a series of carotene-like substances that he summarizes as carotinoids. A specific reaction to carotene is not known. The xanthophil, C_40_H_56_O_2_, is different by having other conditions of solubility and different color (Willstätter and Mieg [109] - about carotene, compare further: Molisch, H. [112] (not seen), Czapek, [113]).

The red pigment of the *Micrococcus rhodochrous *is, according to Zopf [114] and Overbeck [115], insoluble in water, soluble in alcohol (reddish yellow), sulfur carbon (red like a rose), petroleum ether (orange red) etc. It makes blue crystals with concentrated sulfuric acid. The spectrum shows a wide band around the Line F, between 500 and 475 μμ, and is widely shadowed towards G. The color of the cultures corresponds, according to the illustrations of Overbeck, quite to that of the bacteria of the klippfish. With regard to the very similar pigment of the *Micrococcus erythromyxa*, Zopf [116] later states that it is already exuded in the living colonies between the cells in the crystal groups.

The sensation and the excitement that *Bacillus prodigiosus *has called forth (compare [117]) since the ages is probably connected with the fact that this bacterium and its pigment, that Kraft [118] called prodigiosin, have already been studied early and particularly often (compare especially: Schroeter [119], Griffiths [120], Rosenberg [121] , not seen. - Further literature see Scheurlen [117], compare also Flügge, [122]). The pigment is almost insoluble in water, but is soluble in alcohol, ether, acetic acid, xylol, sulfur carbon, etc. The color of the solution changes to carmine red by a little, to violet by more acid, and to yellow by alkali. There are resulting somewhat different spectra, in which essentially two bands were standing out in the green and blue green. Their position according to the not very accurate indications of Scheurlen, Griffiths and Kraft corresponds to the wave lengths 547 to 530 and 501 to 475 μμ.

The red pigment of the often mentioned *Bacillus *of Kiel found in the tap water of Kiel by Breunig [123] is, according to Breunig, soluble in alcohol, chloroform and ether and according to Schneider [124] also in sulfur carbon and benzol, but insoluble in water. It is according to Schneider, bleached by zinc and acetic acid, but restored in air. With potassium it becomes yellow, but with acetic acid red again. The spectrum shows a darkening of about 546 to 540 μμ, a beautiful absorption band from 540 to 528 μμ and darkening from 425 μμ downward (these wavelengths correspond to approaching that of the author's given numbers 63 to 65, 65 to 70 and 135, which clearly refer to the scales of Bunsen and Kirchhoff). These details are also found with Migula [125] under *Bacillus kiliensis*, while Flügge [126] calls the Breunig one *Bacillus B. ruber balticus*. He gives dissenting information about the solubility which apparently in part, are due to the publication of Laurent [127].

According to Petrow [128], *Bacillus subkiliensis *contains a red pigment that likewise is soluble in alcohol, ether, benzol, etc., and is insoluble in water. There is a broad absorption band that lies between 552 and 509 μμ, it is quite sharp between 546 and 513 μm (the author gives the numbers 60 and 79, 63 and 77). With weak acids the pigment becomes carmine red to violet, with strong saltpeter acid at first yellow, then colorless, with potassium or ammonia golden yellow, with acid red again. After bleaching by nascent hydrogen, the oxygen does not restore the color.

A red pigment is also produced by *Bacillus ruber sardinae*, an organism that was found by Bois Saint-Séverin [129] on oil sardines, but that it grows well on potatoes. It turns to yellow by alkali, to red again by acid, is soluble in alcohol, more easily in water, but barely studied in general.

V. Schrötter [130] gives a few short bits of information about a red *Sarcina*-pigment, of *Sarcina aurantiaea *that also occurs in *Staphylococcus pyogenes aureus*. It is a lipoxanthin that with sulfuric acid becomes indigo blue.

The non red pigments of similar behavior, of which among others, Zopf has described a number, may be left out here.

With regard to the relationship of the red pigment of the salt bacteria to the preceding microbial pigments, a concluding verdict cannot be made, because the latter themselves have not been compared enough in detail and are not sharply differentiated in general. It seems, however, that the pigments of the red bacteria do not correspond to any of the mentioned pigments. The pigments of the *Sarcina *and of the *Micrococcus *differ from the others by the fact that they cannot be dissolved at all in any of the common solvents. The pigment of the red *Bacillus *is soluble in a series of solvents, but differs in the absorption bands of its spectrum. It comes the closest, spectroscopically, to that of the pigment of *Bacillus prodigiosus*.

It is very noteworthy that specifically some strong common salt loving or common salt tolerant bacteria and also bacteria living on the same nutrient base next to one another bring forth this type of striking red pigment. We know too little about the metabolic processes and that of the pigments to answer the question, if there is any dependency on common causes. It could not be determined, or if the presence of the common salt has any influence on the pigment production, since the bacteria did not grow at all at lower common salt concentrations. Under all circumstances where the bacteria grew well, they were also red colored: in the light and in the dark, at higher and at lower temperatures, and likewise on the different, up to now, however, only little numerous nutrient bases, on which one succeeded in bringing them to development. As far as the experiments reach, the only factor that seems to have a perceptible influence on the formation of the red pigment is oxygen. It was already referred to above, that the layer in the salt dishes lying under the clay tiles remained colorless, and that the development in fluids at greater heights of the fluid (e.g. in 1 to 2 cm high layer of fish decoction in a test tube) was connected with noticeably little pigment production. But presumably this is simply a consequence of the circumstance that the bacteria do not grow well under oxygen deficiency.

In other pigmented bacteria there have been observed noticeable fluctuations in the pigment production under the influence of external conditions. There were reports by: Schottelius [131], Laurent [132], Galcotti [133], Petrow [128], Milburn [134] regarding pigment loss at higher temperatures, Ritter [135] about the absence of an influence of light, Wasserzug [136], Laurent [132]. Milburn [134], and Knebler [137], on the influence of acid and alkali in the nutrient base. Kuntze [138], Samkow [139], Sullivan [140] about the dependence of pigment production on the presence of magnesium, phosphorus, sulfur, nitrogen. Woolley [141], Ritter [135], Delanoe [142] about the importance and the inhibiting influence of the sugar, Samkow [139], Schottelius [131] about the necessity of the access of oxygen. As particularly convenient for the production of pigment were, according to Didlake [143], soybeans, in other cases, according to Milburn [134], potatoes. By selection and further cultivation of weakly colored colonies there were Schottelius [131] and Hefferan [144] and, through the influence of higher temperatures, it was Laurent [132] who succeeded in obtaining strains which retained the colorlessness, but were not changed with regard to their metabolic products and their other characteristics. Such strains can in general be reconverted into the colored form by changes of the conditions or by selection, and, according to Bertarelli [145], especially also by the passage through animals.

### G. The originator of the coloration of the klippfish

After the preceding examinations it was stated that at least three, perhaps even more quite different salt loving bacterial forms occur on the klippfish that in quite similar ways bring forth noticeably red pigmentation. There remains the question to be answered if one of these forms is exclusively or preferably responsible for the red coloration of the klippfish.

All three bacteria - when taken from the culture and transferred to fish - also bring forth their red color here. This, however, only proves that they also can live on the fish producing red pigments, but it does not easily prove that they are the causative agents for the damaging red coloration; moreover, in order to show this, it would be necessary firstly to prove the concordance of the appearances caused by the bacteria with the red coloration of the fish done in factories and secondly to state the presence of sufficient amounts of the red bacterial forms in question on the red fish. The first evidence provides difficulties because the result of an artificial inoculation and of an artificially supported culture usually gets a somewhat divergent character.

The mentioned peculiarities of the pure culture, the more burnt red or the sharper orange color and the clear oily look of the colonies of the *Bacillus *on the one hand, and the little weaker, more pink-like color and the turbid or somewhat milky look of the colonies of the *Sarcina *and the *Micrococcus *on the other hand, appear even in the culture on the fish. They would, however, fail, if both were present next to one another.

One will have to pay attention to the occurrence of the three bacterial forms on the fish. With regard to this, my studies at the time, when there was still an abundance of fish available for me, had not progressed far enough to be able to differentiate between the bacteria, to split them up and to easily identify them. When the latter was possible, the fish shipments halted due to the political situation. So, for the time being, I am only capable to report about a few probes I got from Cuxhaven the last time when the fish was still available. On one of these probes that I brought in the Fall of 1918, the red coloration consisted almost exclusively of small, isolated, pale red *Sarcina *colonies. They barely expanded, although the fish piece remained permanently damp and was kept in a closed flask in a warm room. One could not state anything about a penetrating of the red coloration into the flesh; it moreover gradually took up a brownish-yellow color. It had a very sharp foul odor, but did not show any particular appearances of degradation, such as turning to liquid manure and the like. Without success, the experiment was done to bring forth other vegetation through the influence of more humidity and higher temperature in the thermostats and to isolate other red bacteria, especially the red *Bacillus*. In this case, the *Sarcina *was at least the essential, if not the only cause of the red coloration of the fish. Also on a second probe, that I obtained in the winter of 1918, the *Sarcina *was the essential cause, and likewise it was the predominant component in the above described red growths on the wall of the storage rooms (January 1919).

In contrast I got the *Bacillus *again out of a probe of a fish that lay before me in December 1918. If I remember right, the fish I had available at the beginning of the studies and from which the red *Bacillus *at first was isolated, had a different appearance and showed smearier, shiny red coatings. I even had to presume that because at first I completely relied upon the belief to have found that the *Bacillus *was the cause of the red coloration. Surely the very first probe that I received had an aberrant appearance, since the flesh seemed to be diffusedly soaked with red color.

The information of the older observers, too, point to the differences in the kind of the red coloration.

I can still say little about the occurrence of the red *Micrococcus*. It seems to live mostly in the company of the *Sarcina *and to be mixed in with their colonies. There is, however, no mutual dependence, since both grow well in pure culture and are always red. If in the future there again will be more abundant shipments of the fish available, the questions with regard to the occurrence of these three and possibly further red bacteria, with regard to their occurrence and the conclusions based on my findings differing somewhat from those of the previous observers, and now, since certain methods for their isolation have been found, will easily be able to be determined.

Questions that ought to be debated afterwards are those with regards to how far the red bacteria are solely responsible for the degradation which causes the reddened klippfish to be disgusting or inedible, to what extent are there still other bacteria involved and what is the nature of the degradations that they bring forth? It is certain that the red bacteria obtain their nutrients from the organic materials present in the klippfish and therefore also cause their degradations. But equally it is doubtless that in addition to the red bacteria there are also other ones not only present on the klippfish, but also able to live on it. On isolating the red bacteria there were repeatedly findings of uncolored or weakly colored ones that grew equally well on the strongly common salt containing nutrient base as the red ones. The *Sarcina *droplets mentioned found on the last mentioned fish were also never completely pure although they mainly consisted of *Sarcina*. The undertaken task however consisted at first, only in stating the cause of the red coloration, and the other bacteria were therefore left out of consideration.

With regard to the practical attempts to fight the bacteria, essentially the same is true as what is said at the end of the section on the *Torula*.

## Competing interests

The authors declare that they have no competing interests.

## Authors' contributions

PD translated the article and assembled it for publication, HK and GK assisted greatly in the translation process. All authors have read and approved the final manuscript.
